# Inequality and barriers in psychosis prevention: A systematic review on clinical high-risk for psychosis studies from developing countries

**DOI:** 10.3389/fpsyt.2023.1148862

**Published:** 2023-04-11

**Authors:** Alexandre Andrade Loch, Ana Caroline Lopes-Rocha, Feten Fekih-Romdhane, Martinus Theodorus van de Bilt, Gonzalo Salazar de Pablo, Paolo Fusar-Poli

**Affiliations:** ^1^Laboratorio de Neurociencias (LIM 27), Faculdade de Medicina, Instituto de Psiquiatria, Hospital das Clinicas HCFMUSP, Universidade de São Paulo, São Paulo, Brazil; ^2^Conselho Nacional de Desenvolvimento Cientifico e Tecnológico, Instituto Nacional de Biomarcadores em Neuropsiquiatria (INBION), São Paulo, Brazil; ^3^Faculty of Medicine of Tunis, Tunis El Manar University, Tunis, Tunisia; ^4^Department of Psychiatry Ibn Omrane, The Tunisian Center of Early Intervention in Psychosis, Razi Hospital, Tunis, Tunisia; ^5^Early Psychosis - Interventions and Clinical-Detection (EPIC) Lab, Department of Psychosis Studies, Institute of Psychiatry, Psychology and Neuroscience, King’s College London, London, United Kingdom; ^6^Department of Child and Adolescent Psychiatry, Institute of Psychiatry, Psychology and Neuroscience, King’s College London, London, United Kingdom; ^7^Child and Adolescent Mental Health Services, South London and Maudsley NHS Foundation Trust, London, United Kingdom; ^8^Department of Child and Adolescent Psychiatry, Institute of Psychiatry and Mental Health, Hospital General Universitario Gregorio Marañón School of Medicine, IiSGM, CIBERSAM, Universidad Complutense, Madrid, Spain; ^9^Department of Brain and Behavioural Sciences, University of Pavia, Pavia, Italy; ^10^Outreach and Support in South London (OASIS) Service, South London and Maudsley NHS Foundation Trust, London, United Kingdom

**Keywords:** schizophrenia, at risk mental state, subclinical psychosis, attenuated psychosis, stigma

## Abstract

**Background:**

The clinical high-risk for psychosis (CHR) paradigm is one of the best studied preventive paradigms in psychiatry. However, most studies have been conducted in high-income countries. It is unclear if knowledge from such countries applies to low and middle-income countries (LAMIC), and if there are specific limitations hindering CHR research there. Our aim is to systematically review studies on CHR from LAMIC.

**Methods:**

A multistep PRISMA-compliant literature search was performed in PubMed and Web of Science for articles published until 1/03/2022, conducted in LAMIC, addressing the concept and correlates of CHR. Study characteristics as well as limitations were reported. Corresponding authors of the included studies were invited to answer an online poll. Quality assessment was done with the MMAT.

**Results:**

A total of 109 studies were included in the review: none from low-income countries, 8 from lower middle-income countries, and 101 from upper middle-income countries. The most frequent limitations were small sample size (47.9%), cross-sectional design (27.1%), and follow-up issues (20.8%). Mean quality of included studies was of 4.4. Out of the 43 corresponding authors, 12 (27.9%) completed the online poll. They cited further limitations as few financial resources (66.7%), no involvement of population (58.2%) and cultural barriers (41.7%). Seventy five percent researchers reported that CHR research should be conducted differently in LAMIC compared to high-income countries, due to structural and cultural issues. Stigma was mentioned in three out of five sections of the poll.

**Discussion:**

Results show the discrepancy of available evidence on CHR in LAMIC, given the shortage of resources in such countries. Future directions should aim to increase the knowledge on individuals at CHR in such settings, and to address stigma and cultural factors that may play a role in the pathways toward care in psychosis.

**Systematic Review Registration:**

https://www.crd.york.ac.uk/prospero/display_record.php?RecordID=316816, CRD42022316816.

## 1. Introduction

The Clinical High-Risk for psychosis (CHR) concept was established three decades ago to identify pre-clinical stages of schizophrenia and to prevent the development of the disorder (1). It has been one of the most well-studied paradigms in psychiatry (2), and much knowledge has been gained through it concerning psychoses’ pathophysiology (3) and psychiatric disease pathways (4, 5). However, there are still some important knowledge gaps on the topic, especially if we consider that most data is generated from high-income countries, and that there is a significant interplay between psychosis and socio-cultural environment (6).

The study of psychosis in different socio-cultural settings dates back to the 70s with the International Pilot Study of Schizophrenia (7). This large multicentric initiative showed that individuals with schizophrenia included in the sample who were living in low-and-middle income countries (LAMIC) had a significantly better outcome and course of the disorder compared to those living in high-income countries (8). This was hypothesized by authors as an effect of the greater tolerance and acceptance of symptomatic patients in LAMIC (9). The debate on the possible effects of socio-cultural factors on the disorder continued in the 90s with the International Study of Schizophrenia (ISoS) studies (10). They confirmed the robustness of the developing versus developed countries’ differences, while also raising harsh critics on possible problems in methodology, like selection bias and diagnostic ambiguities (11). More recent studies continued to exhibit these differences in course and outcome, regardless of participant’s clinical characteristics (12), while others saw the statistically significant difference vanish after controlling their data in a more strict fashion (13). Still in this regard, further evidence associated higher levels of income inequality with an increase in the incidence of schizophrenia (14). The debate on this matter is still open and, most importantly, it shows how socio-cultural factors may shape disease trajectory and pathways to care across the psychosis continuum. Despite the availability of data on schizophrenia from diverse socio-economic backgrounds, it seems that much less data is available concerning the CHR paradigm in low-resource settings.

Previous studies have systematically reviewed data from CHR services around the world, but data from LAMIC included in such studies is scarce or absent. A recent review by Salazar de Pablo et al. (15) aimed to address real-world CHR service characteristics such as service configuration, interventions, and outcomes. Fifty-one services were included, but none of them were from LAMIC. Another systematic review intended to report on transition rates of CHR individuals. Among the 130 included studies, only 8 were from LAMIC (16). Another review wished to report on the global geographical distribution and core characteristics to the level of implementation of CHR services (17). Fifty-one percent of all CHR individuals from included studies’ samples were from Western Europe, and 17% from North America, while only 2.1% were from Africa.

To adequately promote psychosis prevention in LAMIC, it is of utmost importance to construct a knowledge base from CHR research in these countries. This evidence should provide information on possible barriers to the implementation of psychosis prevention programs, and insights on the potential need to adapt the CHR framework to these settings. As such, this review aims to systematically gather data from published CHR studies conducted in LAMIC. We wish to specifically address the question of which are the limitations reported by these studies and what are the barriers toward conducting CHR research in LAMIC. To address this AIM, PICOS (18) criteria were set as follow: (1) Population: CHR individuals living in LAMIC; (2) Intervention: both observational and intervention studies will be included; individuals subjected to clinical evaluation and defined as CHR; (3) Comparison: CHR individuals living in high-income countries; (4) Outcomes: characteristics and outcome of participants, study characteristics; (5) Study: both observational and experimental.

## 2. Methods

This study (study protocol: PROSPERO CRD42022316816) was conducted in accordance with PRISMA (19) (Supplementary Table S1) checklist.

### 2.1. Search strategy and selection criteria

A multistep systematic literature search strategy was used to identify relevant articles by two independent researchers (AAL, MTB). The first search was conducted with Pubmed database (National Institutes of Health). The second search was performed with the Web of Science database (Clarivate Analytics), incorporating the Web of Science Core Collection, KCI-Korean Journal Database, Current Contents Connect, Derwent Innovations Index, and SciELO Citation Index. Both searches included works from inception until 1st March 2022 with no restrictions on language. The following search terms were applied: (“risk” OR “prodrom*” OR “ultra-high risk” OR “clinical high risk” OR “CHR” OR “UHR” OR “attenuat*” OR “high risk” OR “genetic high risk” OR “risk syndrome” OR “at risk mental state” OR “at-risk mental state” OR “ARMS” OR “risk of progression” OR “schizophrenia” OR “schizoaffective disorder” OR “schizophreniform disorder”) AND (“psychosis”). The references of the articles identified in previous reviews and relevant commentaries and the references from the included studies were manually searched to identify additional relevant records. Abstracts were screened, and potential full texts were assessed against inclusion and exclusion criteria.

The inclusion criteria were (a) individual studies, (b) conducted in CHR individuals as defined according to established instruments: Comprehensive Assessment of At-Risk Mental States [CAARMS; (1)], Structured Interview for Psychosis-risk Syndromes [SIPS; (20)], Bonn Scale for the Assessment of Basic Symptoms [BSABS; (21)], Basel Screening Instrument for Psychosis [BSIP; (22)], Schizophrenia Proneness Instrument (23)—Adult (SPI-A) and Child and Youth (SPI-CY) version -, Positive and Negative Syndrome Scale [PANSS; (24)], Scale for the Assessment of Negative Symptoms [SANS; (25)], Brief Psychiatric Rating Scale [BPRS; (26)] and Early Recognition Inventory (ERIraos37), (c) in low- and middle-income countries defines as per the World Bank website,^1^ as accessed in 01/03/2022, (d) in any language.

The exclusion criteria were (a) abstracts, conference proceedings, study protocols, reviews, guidelines, (b) Studies that do not effectively enroll a sample of individuals with the CHR condition, (c) Studies that presume CHR condition by instruments other than the ones described above (e.g., population studies of psychotic-like experiences).

### 2.2. Descriptive measures and data extraction

Independent researchers (AAL, ACLR) extracted data from the included studies; discrepancies were resolved through consensus, consulting a senior researcher (PFP). The variables included (beyond general data such as first author, year of publication, city, and country) were: (i) type of study/study design; (ii) aim of the study; (iii) characteristics of the study: individuals involved in the study, subgroup within CHR, conversion rate, intervention content, assessment tool, recruitment strategy (outreach or referral), (iv) sample characteristics: age, %male, if subjects received psychotropics drugs before entering the study, drug use; (v) key findings; (vi) limitations described by authors in the paper.

Furthermore, an online survey was sent to each of the corresponding author from the articles included in the review, with the following questions: (i) “Besides the limitations cited in your published article, what difficulties have you found in implementing an CHR study in a LAMIC?” Options (multiple choices allowed): few financial resources available; lack of staff research knowledge; no involvement of population; regulatory difficulties; cultural barriers; others—please specify; (ii) “Have you found any difficulties in following-up your CHR cohort?” Yes or no, and “please tell us which difficulties”; (iii) “Have you found any additional difficulty in publishing your paper, compared to other papers you have published?” Yes or no, and “please tell us which difficulties.” (iv) “Based on your knowledge of the CHR literature and on your experience in conducting CHR research in a LAMIC, which one do you think is the best recruitment strategy for this type of research in LAMIC?” Options (multiple choices allowed): Clinic-based/referral: mental health professional referral (e.g., psychiatrist, psychologist); Clinic-based/referral: referral from general practitioners; Clinic-based/referral: referral from community gatekeepers (e.g., religious leaders, community leaders); Outreach/active: population surveys, schools screenings, workshops, service promotion; Outreach/passive: webpages, social media sites; (v) “Do you think CHR research and prevention strategies in LAMIC should be done in a different way compared to developed countries?” Yes or no, and “please tell us which difficulties.”

At last, we reported quality assessment (see below).

### 2.3. Data analysis

All the studies were systematically summarized in tables reporting on various study characteristics (Table 1). We complement this with descriptive analysis of difficulties reported by the corresponding authors who could be contacted (Table 2). An online tool^2^ was used to create a graphical representation of the geographical distribution of the CHR studies included in the review.

**Table 1:** Articles included in the review according to income level.

Country	N (%)	Income level
China	62 (55.4%)	Upper-middle income
Brazil	15 (13.4%)	Upper-middle income
Mexico	7 (6.3%)	Upper-middle income
Turkey	7 (6.3%)	Upper-middle income
Kenya	5 (4.5%)	Upper-middle income
Russia	5 (4.5%)	Upper-middle income
Tunisia	3 (2.7%)	Lower-middle income
India	2 (1.8%)	Lower-middle income
Nigeria	2 (1.8%)	Lower-middle income
Belarus	1 (0.9%)	Upper-middle income
Argentina	1 (0.9%)	Upper-middle income
Malaysia	1 (0.9%)	Upper-middle income
Iran	1 (0.9%)	Lower-middle income

**Table 2:** Characteristics of included articles.

Characteristic	Variable	Value
Sample size	Mean	112.5
	Median	52
	Min-Max	6–600
Study design	Prospective/cohort cross-sectional single-blind randomized trial	44 (39.3%)67 (59.8%)1 (0.9%)
Sociodemographics	Mean age (years)	19.5
	Gender (male)	50.5%
Recruitment strategy^+^	Outreach (n,%)	25 (23.8%)
	Referral (n,%)	80 (76.2%)
Instrument used^++^	SIPS	80%
	CAARMS	15%
	Other	10%
Subtypes of CHR	Studies that reported (n,%)	34 (30.6%)
	APS	93.8%
	BLIPS	5.4%
	GRD	5.1%
Conversion	# of studies (n,%)	43 (38.4%)
	Rate	21.1%
Limitations cited in the articles^+^	Small sample size	48%
	Cross-sectional design	26%
	Follow-up issues	20%
	Single-center study	8%
	Help-seeking strategy	7%
	Not controlled for medication use	5%
	Naturalistic design	5%

+ 7 studies did not report on recruitment strategies, and 2 had mixed strategies.

++ Not mutually exclusive.

### 2.4. Quality assessment

We used the mixed Methods Appraisal Tool (MMAT) (27) questions to assess the quality of the included studies (Supplementary Table S1), considering the content and characteristics of the studies according to our inclusion criteria.

## 3. Results

### 3.1. Summary of search results

**Figure 1:**
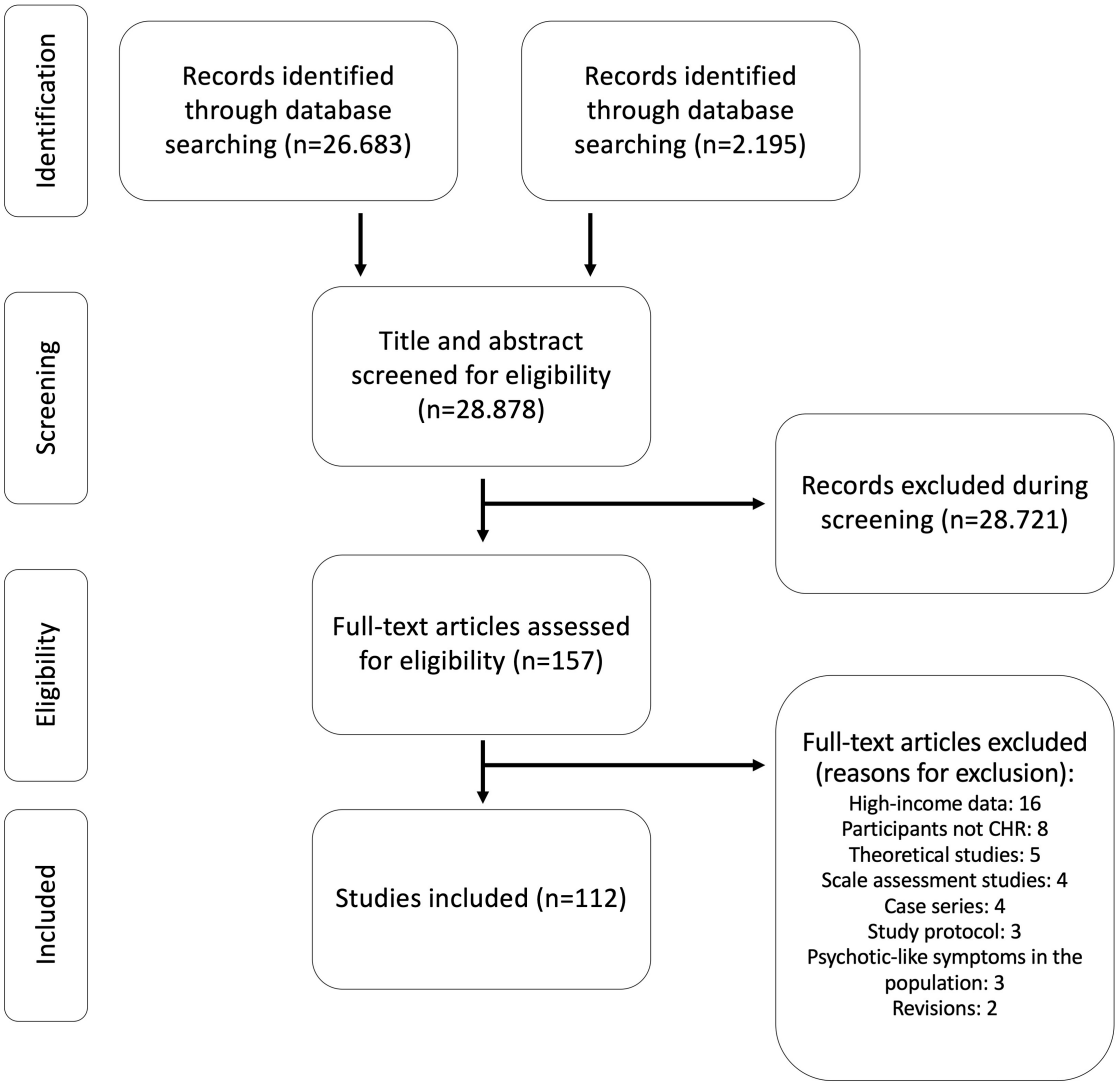
PRISMA flow chart of included studies.

The search strategy generated 28,878 studies (Figure 1). Of these, 157 were selected as potentially relevant and upon full text examination 112 met inclusion criteria. Included articles were from the following 13 countries: China (*n* = 62, 55.4%), Brazil (*n* = 15, 13.4%), Mexico (*n* = 7, 6.3%), Turkey (*n* = 7, 6.3%), Kenya (*n* = 5, 4.5%), Russia (*n* = 5, 4.5%), Tunisia (*n* = 3, 2.7%), India (*n* = 2, 1.8%), Nigeria (*n* = 2, 1.8%), Belarus (*n* = 1, 0.9%), Argentina (*n* = 1, 0.9%), Malaysia (*n* = 1, 0.9%), and Iran (*n* = 1, 0.9%) (Table 1).

### 3.2. Characteristics of the included studies

A total of 12,602 CHR assessments were made within the included studies. Mean sample size was of 112.5 participants (6–600), with a median of 52 participants (Table 2). Most studies were cross-sectional (59.8%), and only 1 was an intervention study. Mean age was of 19.5 years, and pooled percentage of males was of 50.5%. Outreach strategy was used in 25 (23.8%) studies, while referral was used in 80 (76.2%) studies. The SIPS was used in 80% of the studies, the CAARMS in 15%, and other scales were used in 10% (e.g., BPRS, Prodromal Questionnaire). Pooled percentage of CHR sub-syndromes was of 93.8% APSS, 5.4% BLIPS, and 5.1% GRD. Transition to psychosis was assessed in 43 studies (38.4%), and transition rate to psychosis was of 21.1%.

### 3.3. Limitations described in the studies and quality assessment

As for the limitations described in the publications, 48% papers cited small sample size as a major limitation. Cross-sectional design (and lack of follow-up data) and follow-up issues (attrition, difficulties in recalling participants) was reported by 26 and 20% of the studies, respectively. The fact that it was a single-center study (8%), help-seeking strategy (7%), medication use as a confounding factor (5%), and naturalistic design (5%) were other commonly cited issues. Overall mean quality score according to the MMAT was of 4.3.

### 3.4. Online poll sent to corresponding authors

Regarding the online poll, of the 43 corresponding authors, 12 completed the survey (27.9%). Besides the limitations described in their published papers, described in the section above, 66.7% reported few financial resources, 58.3% cited that there was no involvement of the population, and 41.7% stated cultural barriers as further limitations to the conduction of the CHR study (Table 3). Difficulties in following-up their cohort was stated by 77.8% of the authors. All the commentaries for this question described patients’ reluctancy to participate in the longitudinal assessments. Difficulties in publishing their work was reported by 25% of authors. Small sample size and lack of follow-up data were the main reasons reported for this.

**Table 3:** Results of the online poll with corresponding authors.

Question	Options given	Value	Comments
Besides the limitations cited in your published article, what difficulties have you found in implementing a CHR study in a LAMIC?^+^	- Few financial resources available - No involvement of population - Cultural barriers - Lack of staff research knowledge - Regulatory difficulties (ethics committee, etc.) - Other: Stigma Recruitment issues	66.7% 58.3% 41.7% 25% 8.3% 8.3% 8.3%	—
Have you found any difficulties in following-up your CHR cohort?	- Yes - No	77.8% 22.2%	- “Participants never show up again” - “People refusing to be re-evaluated” - Difficult to maintain long-term follow-up (patients from all over the country); “research team lacks stability and there are insufficient researchers.” - Patients did not return to the clinic for follow-up. Immigrant participants. “Trained clinical assessment staff also have demanding clinical duties and little time for research work” - Hard to engage people with mild symptoms in a long follow-up - Rejection rate is high for face-to-face interviews
Have you found any additional difficulty in publishing your paper, compared to other papers you have published?	- Yes - No	25% 75%	- Lack of power due to small sample size - Small sample size, lack of follow-up data, difficult to publish in high-quality journals
Based on your knowledge of the CHR literature and on your experience in conducting CHR research in a LAMIC, which one do you think is the best recruitment strategy for this type of research in LAMIC? (multiple choices allowed)^+^	- Clinic-based/referral: mental health professional referral (e.g., psychiatrist, psychologist) - Outreach / active: population surveys, schools screenings, workshops, service promotion - Clinic-based / referral: referral from general practitioners	75% 41.7% 25%	- (outreach) “circumvents cultural barriers.” - People with mental disorders are stigmatized, “therefore CHR with medical need are more likely included in the studies.” - (clinic-based approach) “gives a ‘filter’ to other pathologies that share symptoms with CHR syndrome.” - Clinic-based: few subjects but most of them were included. Community gate keepers: lots of referrals but very few subjects were eligible - Recruitment with clinic-based approaches select help-seeking individuals, which is easier, but not necessarily better. They are a specific, but biased sample. Going to the community has scientific advantages, but requires financial support, trained staff, etc.
Do you think CHR research and prevention strategies in LAMIC should be done in a different way compared to developed countries?	- No - Yes	25% 75%	- Less access to formal education - Less clinic-based, involvement of government - Consider task-shifting, giving limited resources - Mental health literacy and stigma affect evaluation of this group - Differences in the role of family, drug use profile between countries, laws regarding autonomy But, all of the basics need to be in place such as validated screening and assessment instruments, translated into the local language, relationships with primary care physicians and/or schools and university, non-stigmatizing interventions, support for families, and community education. Also, support from the Ministry of Health

+ Not mutually exclusive.

**Figure 2:**
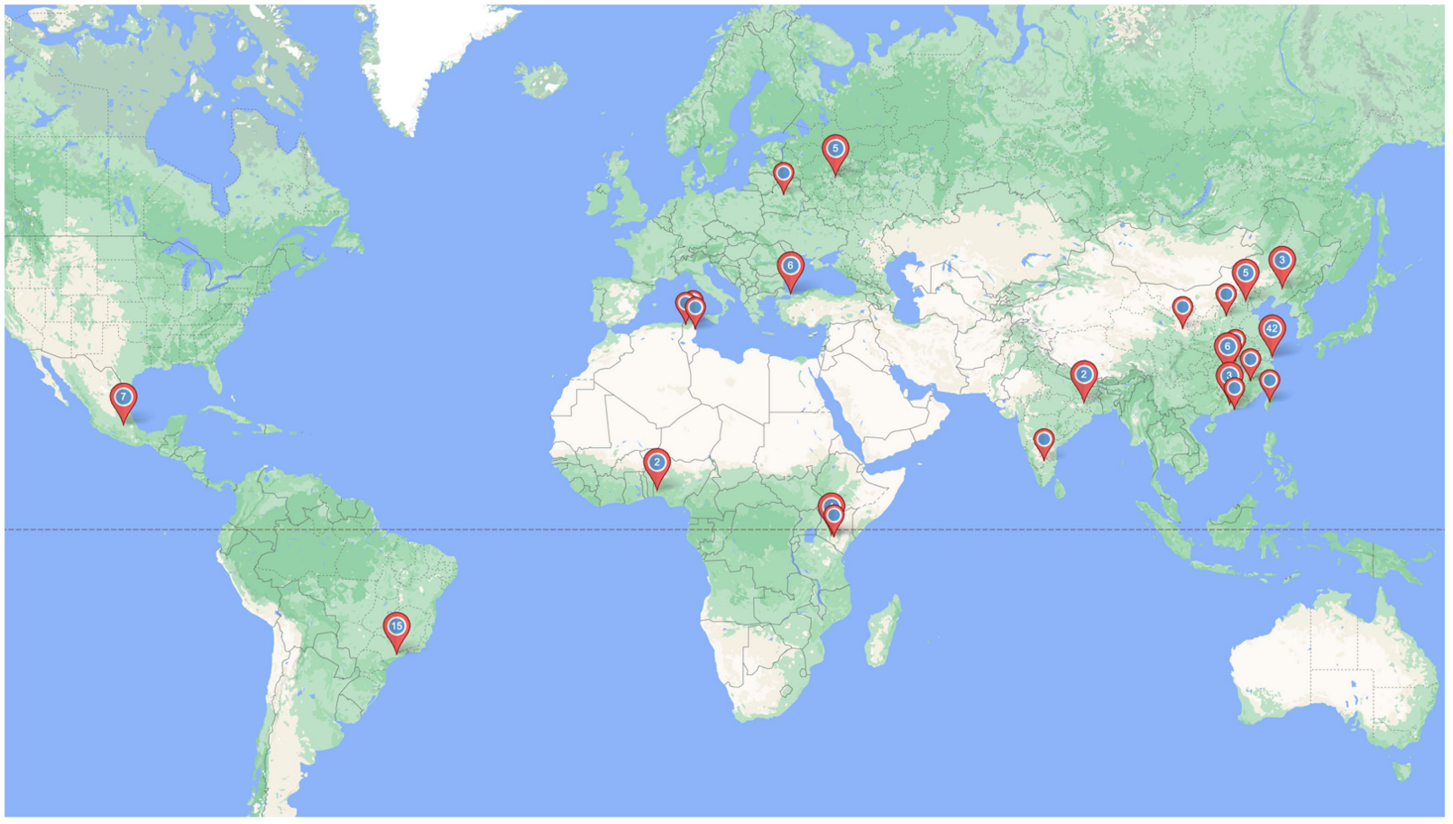
Map of included LAMIC studies on CHR.

Based on their experience in conducting CHR research in a LAMIC, 75% of authors pointed clinic-based and referral by a mental health professional as the desirable CHR approach strategy; 41.7% cited active outreach (population surveys, schools screenings, workshops, service promotion) as another possible approach. And 25% cited clinic-based and referral from community gatekeepers (e.g., religious leaders, community leaders) as a good approach. In the commentaries, stigma and cultural barriers were factors that influenced the choice for outreach strategies. However, for this strategy authors cited that more resources are required (“financial support, trained staff”), and more individuals are screened out (“community gate keepers: lots of referrals but few subjects were eligible”). On the other hand, clinic-based approaches are thought to be more specific (“gives a ‘filter’ to other pathologies”), despite recruiting less individuals and being a more biased sample (Figure 2).

CHR research and prevention strategies in LAMIC should be done in a different way compared to developed countries for 75% of the assessed authors. Structural issues were reported like the need of more involvement of local government, and staff task shifting given human resource limitations. Cultural differences related to mental health literacy and stigma, drug use profile across countries, less access to formal education, and role of families were cited.

## 4. Discussion

### 4.1. General findings

To the best of our knowledge, this is the first study to systematically address published studies on the CHR paradigm in developing countries. The main finding of this review concerns the disparity that follows a parallel between the number of included studies and countries’ income-level. The most frequent limitations mentioned in the publications were small sample size, cross-sectional design, and follow-up issues. As for the online poll with corresponding authors, further limitations as few financial resources, cultural barriers, and no involvement of population were the most frequently cited ones. Most researchers reported having difficulties in following up with their cohort, and that CHR research should be done differently in LAMIC compared to high-income countries. Stigma was recurrently mentioned as an issue throughout the online poll.

Findings can be thus summarized into three topics: disparity in data and funding, follow-up issues, and the cultural barrier/stigma topic.

First, regarding the disparity on the availability of data from LAMIC, no studies from low-income countries were included, 8 studies from lower middle-income countries were included, and the remaining 104 included studies were from upper middle-income countries. To exemplify this inequality, a quick search for a single high-income CHR program on PubMed (inputting the term “North American prodrome longitudinal study,” the NAPLS, one of the most well-established psychosis prevention programs, based in the North America) returned 86 results. Thus, despite that 84.1% of the world population lives in LAMIC (28), few data regarding the CHR framework is generated from these countries.

Establishing a specialized CHR service and conducting research on this topic is not simple and requires adequate funding and dedicated personnel. A recent systematic review by Kotlicka-Antczak et al. (17), including CHR services from all over the world, observed that among the three main challenges affecting these services were lack of financial support (51.1% of services) and inadequate staffing resources (42.6%). This resonates with our findings, as complaints by corresponding authors that participated in the poll were very similar (e.g., “research team lacks stability”). As a matter of fact, these issues should be even worse in LAMIC due to a generalized underfunding of mental healthcare, with a consequent suboptimal number of professionals and services negatively impacting regular mental healthcare delivery (29–31). As a consequence, another possible impact of insufficient funding may be the capacity to recruit and follow-up large samples. While mean sample size found in this review was rather large, median size was small—about half of the mean value. This indicates that only a few studies, mainly from China, were larger and enrolled >100 individuals. This also resonates with the main limitation cited in half of the included publications, namely of small sample size.

Despite the lack of resources, overall characteristics of the included studies were similar to those reported in the international literature on CHR. In a systematic review and meta-analysis to assess the probability of transition to psychosis among CHR individuals (16), mean age of individuals of the 130 included studies (mostly from high-income countries) was of 20.3 years, against 19.5 in ours. In another systematic review on CHR services, this time only including high-income countries, the proportion of male participants in the 49 included studies was of 57.2%, slightly higher than our pooled proportion (50.5%) (15). As for CHR subtypes, the pooled APS percentage observed (93.8%) was somewhat higher than that described in the referred systematic review of high-income countries CHR services (82.6%) (15). Also compared to this publication, BLIPS (5.5% in them vs. 5.4% in ours) were at a similar proportion and GRD (8.0% vs. 5.1%) proportion was slightly lower in our results. At last, mean conversion rate observed in the current study (21.1%) was somewhat lower compared to the meta-analytical work on the probability of transition (25%) (16). Consequently, data shown here suggests convergent validity of the CHR research paradigm, even though funding to conduct these studies is unevenly distributed.

### 4.2. Difficulties in gathering longitudinal data

The second issue that draws our attention is the difficulty in engaging CHR individuals. This was another main limitation reported in the included studies (cross-sectional design and follow-up issues) and was also a concern for most authors that responded the poll (“no involvement of population,” problems in following-up the cohort). Difficulty in enrolling subjects has been reported as the main barrier toward the implementation of CHR services (declared by 53.2% of participating services), according to the previously cited review by Kotlicka-Antczak et al. (17). Furthermore, a recent review by Beck et al. on the outcome of CHR individuals displays attrition rates as high as 52% in the follow-up of these subjects (32). One main factor that affects follow-up data also regards the previous topic of insufficient funding, as longitudinal studies are much more resource-consuming than cross-sectional ones. According to corresponding authors, “research team lacks stability and there are insufficient researchers,” “trained clinical assessment staff also have demanding clinical duties and little time for research work.” Another factor that may hamper the follow-up of CHR cohorts is discussed as follows, namely stigma and cultural barriers.

### 4.3. Cultural barriers and stigma

The cultural barrier/stigma issue appears as the third main topic of this review’s results. Cultural barrier was the third most frequently cited limitation by corresponding authors. Stigma was among the factors that should guide a different CHR research strategy in LAMIC as compared to high-income countries, according to authors (“People with mental disorders are stigmatized,” “Mental health literacy and stigma affect evaluation of this group,” need for “non-stigmatizing interventions”). In this sense, a wide array of published works shows that stigma toward psychosis and low mental health literacy—one of stigma’s manifestations—constitutes a major problem in developing countries. For instance, recognition of mental disorders is low in China according to a nationwide study published in 2019, with psychosis/schizophrenia having the lowest recognition level (33). In Brazil, another nationwide study showed that the recognition of a schizophrenia vignette by general population participants was associated with stigmatizing beliefs on the disorder (34). Surprisingly, mental health professionals included in the study were the ones who presented the most stigmatizing beliefs toward people with the disorder. In Turkey, a systematic search of the national literature showed that healthcare providers, caregivers and families, the public and students had negative attitudes toward people with schizophrenia (35). Additionally, stigma also contaminates the pre-clinical stages of psychosis, as further evidence drawn from a systematic review showed that there is also stigma associated with the CHR label (36). As such, the combination of low mental health literacy and negative beliefs and attitudes toward psychosis may have impacted subjects’ willingness to participate in CHR studies, generating the referred difficulties in recruitment and follow-up (36, 37).

Indeed, stigma is pointed out as the main reason that hampers the contact with health services all along the psychosis spectrum: from adherence in those diagnosed with psychotic disorders (38), to increased duration of untreated psychosis and delay in early identification in those without a formal diagnosis (39). Stigma toward psychosis is an universal phenomenon, but the overall paucity of data on the matter in LAMIC worsens its negative impact and fails to provide evidence to anti-stigma initiatives in these countries (40).

Stigma, on its turn, is also intertwined with the scarcity of resources and with cultural aspects, importantly affecting help-seeking in psychosis in these settings (30). Because of these, pathways toward care in psychosis in LAMIC is often different compared to high-income countries (41). A systematic review on the issue found that a large proportion of patients with psychosis use religious healers as their first point of contact for accessing care (41). Psychotic symptoms are therefore frequently interpreted by the leigh belief as supernatural or religious manifestations—as shown by previous works in Brazil (42), Kenya, Nigeria, Ghana (43), and many other countries, for instance (42, 44, 45). This also occurs in individuals with an Islamic background, who may attribute psychotic symptoms to *jinn* (invisible spirits) (46). Accordingly, religion may have an ambiguous role in psychosis: in some occasions it may offer an important tool to contextualize and cope with psychotic experiences (47, 48). On the other hand, this framework, present in many LAMIC, may significantly increase the duration of untreated psychosis, and implies in a potential barrier to early identification and intervention (49, 50). Cultural aspects and stigma may constitute a barrier toward mental health service usage even in communities of individuals from LAMIC-descent cultures living in developed countries. This has been shown in a study enrolling Mexican-descent families of individuals with serious mental disorders living in a large city in the Southwest United States, for instance (51).

Accordingly, corresponding authors contacted in this review acknowledged the need to adapt CHR programs run in high-income countries to LAMIC. As an example, researchers in Ghana and Nigeria established a collaborative framework between traditional faith healers and conventional health-care providers to address people with psychosis (52). In their cluster randomized controlled trial, Gureje et al. observed that this approach was effective and cost-effective, circumventing the lack of mental health services and professionals and harmonizing scientific-based intervention with local cultural practices and beliefs. Also, alternative and cost-sparing ways of screening for psychosis should also be employed, given the shortages of resources (53). Another cost-sparing approach that would be suited for LAMIC concerns the use of speech-based techniques to screen CHR individuals. This has been described in a work by Argolo et al. (53), in which a simulation of a perfect screening software based on language analysis would spare USD 9.34 billion for the healthcare system of Maputo, Mozambique.

### 4.4. Limitations

Our results should be interpreted considering some limitations. First, a small number of corresponding authors participated in the online survey. Though the survey was very brief, lasting a few minutes, only around one third of corresponding authors responded to our e-mails. This may be explained by the lack of time and resources, as task-shifting, for instance, was cited by authors as one of the problems concerning staff. Another reason may be that researchers shifted their research focus, in the sense that many contacted authors are not studying CHR anymore, according to their recent published works. Second, most studies were observational, only one intervention study was found and no randomized controlled trials, so the efficacy of interventions and other therapeutic factors remain unknown in LAMIC. This limitation is inherent to CHR and is observed at a meta-analytical level (54). The third limitation concerns the stricter inclusion criteria of the revision concerning types of work. Some conference papers were seen in the review process which apparently never turned into a published article, and thus were excluded by this review’s criteria. Also, the review did not include gray literature, thesis, and other sorts of literature. So, there might be more available data on CHR in LAMIC that failed to be published in regular journals. Other limitations may include lack of studies on specific denominations [e.g., APS (55), BLIPS/BPE (56), adolescents (57)], and biomarkers [e.g., neurocognition (58)]. Some studies do not include in their description that they are assessing a CHR sample. On the other hand, including all the specific denominations and biomarkers would render the review unfeasible.

## 5. Conclusion

This systematic review showed the discrepancy of available evidence on CHR in LAMIC, given the shortage of resources in such countries. Therefore, efforts should be taken in order to increase the knowledge based on psychosis prevention in such settings. More specifically, future research should address stigma and cultural factors that may play a major role in the pathways toward care in psychosis. The study of these factors should significantly add to the understanding of subclinical psychotic states in different types of environments, and aid in the adaptation of current prevention frameworks to LAMIC.

## Data availability statement

The original contributions presented in the study are included in the article/Supplementary material, further inquiries can be directed to the corresponding author/s.

## Author contributions

AL, GS, and PF-P designed the study. AL, AL-R, and MB collected the data and performed the analysis. AL drafted the manuscript. All authors reviewed and approved the final version of the manuscript.

## Funding

This research was supported with grant from the Wellcome Trust (grant# 223139/Z/21/Z). The funder had no role in the design and conduct of the study; collection, management, analysis, and interpretation of the data; preparation, review, or approval of the manuscript; and decision to submit the manuscript for publication.

## Conflict of interest

The authors declare that the research was conducted in the absence of any commercial or financial relationships that could be construed as a potential conflict of interest.

## Publisher’s note

All claims expressed in this article are solely those of the authors and do not necessarily represent those of their affiliated organizations, or those of the publisher, the editors and the reviewers. Any product that may be evaluated in this article, or claim that may be made by its manufacturer, is not guaranteed or endorsed by the publisher.

## Footnotes

^1^ https://data.worldbank.org/

^2^ https://www.maptive.com
