# Supplementary material for: Inequality and barriers in psychosis prevention: A systematic review on clinical high-risk for psychosis studies from developing countries
Source: Front Psychiatry. 2023 Apr 11;14:1148862. doi: 10.3389/fpsyt.2023.1148862 (PMC10126325; doi:10.3389/fpsyt.2023.1148862)
Supplement: Supplementary file 1 [file Table_1.DOCX]

**Supplementary material**

**Table S1: Prisma statement and checklist**

| **Section/topic** | **#** | **Checklist item** | **Reported**  **in page** |
| --- | --- | --- | --- |
| **TITLE** | | |  |
| Title | 1 | Identify the report as a systematic review, meta-analysis, or both. | 1 |
| **ABSTRACT** | | |  |
| Structured summary | 2 | Provide a structured summary including, as applicable: background; objectives; data sources; study eligibility criteria, participants, and interventions; study appraisal and synthesis methods; results; limitations; conclusions and implications of key findings; systematic review registration number. | 2 |
| **INTRODUCTION** | | |  |
| Rationale | 3 | Describe the rationale for the review in the context of what is already known. | 3 |
| Objectives | 4 | Provide an explicit statement of questions being addressed with reference to participants, interventions, comparisons, outcomes, and study design (PICOS). | 3-4 |
| **METHODS** | | |  |
| Protocol and registration | 5 | Indicate if a review protocol exists, if and where it can be accessed (e.g., Web address), and, if available, provide registration information including registration number. | 4 |
| Eligibility criteria | 6 | Specify study characteristics (e.g., PICOS, length of follow-up) and report characteristics (e.g., years considered, language, publication status) used as criteria for eligibility, giving rationale. | 4-5 |
| Information sources | 7 | Describe all information sources (e.g., databases with dates of coverage, contact with study authors to identify additional studies) in the search and date last searched. | 4-5 |
| Search | 8 | Present full electronic search strategy for at least one database, including any limits used, such that it could be repeated. | 4 |
| Study selection | 9 | State the process for selecting studies (i.e., screening, eligibility, included in systematic review, and, if applicable, included in the meta-analysis). | 4-5 |
| Data collection process | 10 | Describe method of data extraction from reports (e.g., piloted forms, independently, in duplicate) and any processes for obtaining and confirming data from investigators. | 4-5 |
| Data items | 11 | List and define all variables for which data were sought (e.g., PICOS, funding sources) and any assumptions and simplifications made. | 5 |
| Risk of bias in individual studies | 12 | Describe methods used for assessing risk of bias of individual studies (including specification of whether this was done at the study or outcome level), and how this information is to be used in any data synthesis. | 5 |
| Summary measures | 13 | State the principal summary measures | 5 |
| Risk of bias across studies | 15 | Specify any assessment of risk of bias (i.e. Newcastle-Ottawa Scale (NOS), that may affect the cumulative evidence. | 5, S2 |
| Additional analyses | 16 | Describe methods of additional analyses (e.g., sensitivity or subgroup analyses, meta-regression), if done, indicating which were pre-specified. | N.A. |
| **RESULTS** |  |  |  |
| Study selection | 17 | Give numbers of studies screened, assessed for eligibility, and included in the review, with reasons for exclusions at each stage, ideally with a flow diagram. | 6, figure 1 |
| Study characteristics | 18 | For each study, present characteristics for which data were extracted (e.g., study size, PICOS, follow-up period) and provide the citations. | 20, Table S2 |
| Risk of bias within studies | 19 | Present data on risk of bias of each study and, if available, any outcome level assessment (see item 12). | 20, Table S2 |
| Results of individual studies | 20 | For all outcomes considered (benefits or harms), present a summary data for each intervention group. | 20, Table S2 |
| Synthesis of results | 21 | Present results of study analysed | 6-9 |
| Risk of bias across studies | 22 | Present results of any assessment of risk of bias across studies (see Item 15). | 9 |
| Additional analysis | 23 | Give results of additional analyses, if done (e.g., sensitivity or subgroup analyses, meta-regression [see Item 16]). | N.A. |
| **DISCUSSION** |  |  |  |
| Summary of evidence | 24 | Summarize the main findings including the strength of evidence for each main outcome; consider their relevance to key groups (e.g., healthcare providers, users, and policy makers). | 10-12 |
| Limitations | 25 | Discuss limitations at study and outcome level (e.g., risk of bias), and at review-level (e.g., incomplete retrieval of identified research, reporting bias). | 12 |
| Conclusions | 26 | Provide a general interpretation of the results in the context of other evidence, and implications for future research. | 13 |
| **FUNDING** |  |  |  |
| Funding | 27 | Describe sources of funding for the systematic review and other support; role of funders for the systematic review. | 13 |

**Table S2: Characteristics of the included studies**

|  | **Title** | **First author** | **Year** | **City** | **Country** | **Aim of the study** | **Individuals involved in the study** | **subgroup within ARMS (%)** | **Conversion rate (n,%)** | **Assessment tool** | **outreach/ referral** | **Mean age** | **%male** | **key findings** | **limitations** | **MMAT** |
| --- | --- | --- | --- | --- | --- | --- | --- | --- | --- | --- | --- | --- | --- | --- | --- | --- |
| 1 | Cognitive Patterns and Conversion in a Representative Sample of Individuals at Risk for Psychosis | Haddad NM^1^ | 2022 | São Paulo | Brazil | Relationship between cognition and conversion (CHR x controls) | 92 | N/A | 15 (16.3%) | SIPS | Outreach | 24.9 | 0.39 | Specific cognitive profile was associated with conversion | Small sample size | 5 |
| 2 | Longitudinal and cross-sectional validation of the WERCAP screen for assessing psychosis risk and conversion | Mamah D^2^ | 2022 | Nairobi | Kenya | Compare screening tool (WERCAP) against SIPS | 118 | 100% APSS | 5 (4.2%) | SIPS | Outreach | 19.8 | 0.472 | Excellent validity for the p-WERCAP in screening risk for psychosis (validated by SIPS) | Findings might be culture specific, screening tool not suitable for diagnosis or prediction (WERCAP) | 5 |
| 3 | Neurocognitive Assessments Are More Important Among Adolescents Than Adults for Predicting Psychosis in Clinical High Risk | Zhang T^3^ | 2022 | Shangai | China | compare cognition between adults x adolescents CHR | 325 | N/A | 0.237 | SIPS | Referral | 18.9 | 0.46 | different cognitive performance among groups | single site, naturalistic, possible cultural bias, intelligence not assessed | 3 |
| 4 | Plasma metabolic alterations and potential biomarkers in individuals at clinical high risk for psychosis | Li Z^4^ | 2022 | Shangai | China | Metabolomics for potential biomarkers (CHR x controls) | 90 | N/A | 23 (25.6%) | SIPS | Referral | 18.1 | 0.578 | 104 metabolites found in converters | Small sample size, biomarkers should be validated, antipsychotic treatment might induce conversion | 4 |
| 5 | Plasmatic endocannabinoids are decreased in subjects with ultra-high risk of psychosis | Joaquim HPG^5^ | 2022 | São Paulo | Brazil | quantify plasma cannabinoid levels in CHR x controls | 91 | N/A | 15 (16.5%) | SIPS | Outreach | 24.9 | 0.406 | Lower AEA and 2AG+1AG in CHR x controls | Endocannabinoids assessed in peripheral blood, no anthropometric data | 5 |
| 6 | Baseline Cortical Thickness Reductions in Clinical High Risk for Psychosis: Brain Regions Associated with Conversion to Psychosis Versus Non-Conversion as Assessed at One-Year Follow-Up in the Shanghai-At-Risk-for-Psychosis (SHARP) Study | Del Re EC^6^ | 2021 | Shanghai | China | Investigate primary language regions | 152 | N/A | 0.145 | SIPS | Referral | 18.8 | 0.611 | Fronto-temporo-parietal abnormalities characterize CHR | Small sample size, age was cross-sectional, early follow-up | 4 |
| 7 | Calculating individualized risk components using a mobile app-based risk calculator for clinical high risk of psychosis: findings from ShangHai At Risk for Psychosis (SHARP) program | Zhang T^7^ | 2021 | Shanghai | China | Develop and validate the predictive accuracy and individualized risk components of a mobile app-based psychosis risk calculator (RC) | 300 | N/A | 51 (26.0% - from followed) | SIPS | Referral | 19.1 | 0.48 | The SHARP-RC showed good discrimination of subsequent transition to psychosis | Limited ability to explain the logic underlaying the resulting algorithm,a single center sample recruitment, only 2 years follow-up, underestimation of the cllinical severity of their sample given the lost of participants with higher symptom | 5 |
| 8 | Changes in the cognitive function of Chinese college students with a clinical high risk of psychosis | Luo X^8^ | 2021 | Wuhan | China | effect of cognitive changes on psychosis development (CHR x controls) | 115 | 86% APSS, 13% BIPS, 1% GRD | 29 (27.1%) | SIPS | N/A | 21.3 | 0.476 | Impaired cognitive functioning of CHR x controls; CHR+ are more impaired | Cognition evaluated only at enrollment | 5 |
| 9 | COX-2 pathway is upregulated in ultra-high risk individuals for psychosis | Pereira CAC^9^ | 2021 | São Paulo | Brazil | quantify plasma inflammatory levels in CHR x controls | 55 | N/A | N/A | SIPS | Outreach | 23.5 | 0.418 | PGE2 and TxB2 increased in CHR | N/A | 5 |
| 10 | Enhancing attention and memory of individuals at clinical high risk for psychosis with mHealth technology | Li H^10^ | 2021 | Shangai | China | Effect of app in memory and attention | 80 | N/A | NDA | SIPS | Referral | 20.5 | 0.487 | Mobile technology improved cognition in CHR subjects | N/A | 4 |
| 11 | Establishing a clinical high-risk program in Tunisia, North Africa: A pilot study in early detection and identification | Ventura J^11^ | 2021 | Tunis | Tunisia | Feasibility of a CHR program | 6 | 100% APSS, 17% GRD | 0.17 | CAARMS | Referral | 17.6 | 1 | CHR program in Tunisia is feaseble | N/A | 4 |
| 12 | Further evidence that antipsychotic medication does not prevent long-term psychosis in higher-risk individuals | Zhang T^12^ | 2021 | Shangai | China | Effects of antipsychotic use | 300 | N/A | 56 psychosis (26.7%). 61 (29%) other | SIPS | Referral | 18.7-19.8 | N/A | antipsychotic does not prevent conversion | observational design, heterogeneity of treatment group, no data on concomitant psychological treatment, single-center | 4 |
| 13 | Impaired Sensorimotor Gating Using the Acoustic Prepulse Inhibition Paradigm in Individuals at a Clinical High Risk for Psychosis | Bo Q^13^ | 2021 | Beijing | China | Prepulse inhibition differences CHR x first episode schizophrenia x controls | 55 | N/A | NDA | SIPS | N/A | 21.4 | 0.58 | CHR had similar results to schizophrenia, and different from controls | groups not matched for age and IQ, small sample size, lack of specificity of tested paradigm | 4 |
| 14 | Increased PLA(2) activity in individuals at ultra-high risk for psychosis | Talib LL^14^ | 2021 | São Paulo | Brazil | Serum biomarker | 85 | N/A | 0.176 | SIPS | Outreach | 24.9 | 0.365 | increased PLA2 in CHR | N/A | 5 |
| 15 | Individuals at ultra-high risk of psychosis and first-degree relatives of patients with schizophrenia experience impaired family functionality and social support deficit in comparison to healthy controls | He XY^15^ | 2021 | Guangdong | China | Compare family function and social support FEP, CHR, first-degree relatives of SCZ, HC | 40 | 95% APSS, 5% BIPS | N/A | SIPS | Outreach | 29.1 | 0.4 | CHR family adaptability, family cohesion, and social support reduced relative to HC subjects, but increased relative to FEP | cross-sectional, small sample size | 4 |
| 16 | Influence of migration on the thought process of individuals at ultra-high risk for psychosis | Nogueira AS^16^ | 2021 | São Paulo | Brazil | Migration and symptoms in CHR | 42 | N/A | NDA | SIPS | Outreach | 24.8 | 0.28 | migration influences thougth process in CHR | small sample size, cross-sectional design | 4 |
| 17 | Neurocognition in Kenyan youth at clinical high risk for psychosis | Mamah D^17^ | 2021 | Nairobi | Kenya | assess neurocognition in CHR | 47 | N/A | NDA | SIPS | Outreach | 21.4 | 0.532 | significant cognitive impairment in CHR x controls | cross-sectional, cognitive battery designed for adolescents | 4 |
| 18 | Relationship of negative symptom severity with cognitive symptoms and functioning in subjects at ultra-high risk for psychosis | Uçok A^18^ | 2021 | Istambul | Turkey | Relationship between cognition, negative symptoms and functioning | 107 | 76.4% APSS, 15.1% BIPS, 8.5% GRD | NDA | BPRS-E and CAARMS | Referral | 20.5 | 0.729 | Negative symptoms were associated with deficits in cognitive performance and global and role functioning | Tertiary center enrollment, CHR based on BPRS, selection bias (attrition), GAF for fucntioning, cross-sectional data, no account for other medications such as antipsychotics | 3 |
| 19 | Salivary Metabolomics Reveals that Metabolic Alterations Precede the Onset of Schizophrenia | Cui G^19^ | 2021 | Shangai | China | Salivary metabolomics on HC x CHR x FES | 43 | N/A | N/A | SIPS | Referral | 17.4 | 0.714 | metabolic pathways altered in CHR and FES against controls | N/A | 4 |
| 20 | Screening of the college students at clinical high risk for psychosis in China: a multicenter epidemiological study | Wu J^20^ | 2021 | Shanghai | China | Investigate screening method | 72 | 1.4% GRD, 98.6% APSS | NDA | SIPS | Outreach | 18.3 | 0.472 | 3-stage screening is effective, but rates in general population are low | telephone assessment, incomplete information from participants | 5 |
| 21 | Subtypes of Clinical High Risk for Psychosis that Predict Antipsychotic Effectiveness in Long-Term Remission | Zhang T^21^ | 2021 | Shangai | China | Identify symptom subtypes' responses to antipsychotics | 289 | N/A | 54 (18.7%) | SIPS | Outreach |  |  | Symptom subtypes displayed different trajectories and responses to antipsychotics | No description on AP side-effect and tolerability, accuracy of self-reports, ethnicity bias | 5 |
| 22 | Temporal and time-frequency features of auditory oddball response in distinct subtypes of patients at clinical high risk for psychosis | Wu G^22^ | 2021 | Shangai | China | P300 in three clusters of CHR | 104 | N/A | N/A | SIPS | Referral | 17.6 | 0.51 | cluster 1, who converts, has diminished P300 response | single center, small sample size, lack of control sample, no follow-up | 4 |
| 23 | A Weighted Gene Co-expression Network Analysis Reveals lncRNA Abnormalities in the Peripheral Blood Associated With Ultra-High-Risk for Psychosis | Ren Y^23^ | 2020 | Shanxi | China | WGCNA analysis of IncRNAs | 14 | N/A | NDA | SIPS | N/A | 16.1 | 0.64 | IncRNA modules associated with CHR | small sample size, confounding factors | 4 |
| 24 | Altered cerebellocerebral structural covariance in individuals with attenuated psychosis syndrome | Pu C^24^ | 2020 | Peking | China | Whole brain gray matter abnormalities CHR x HC | 21 | 100% APSS | NDA | SIPS | Referral | 21.6 | 0.66 | Striatal, prefrontal and cerebellar structural changes in CHR vs. HC | Small sample size, only two cerebellar regions investigated, cross-sectional, no psychotic individuals recruited | 4 |
| 25 | An imaging-based risk calculator for prediction of conversion to psychosis in clinical high-risk individuals using glutamate (1)H MRS | Kegeles LS^25^ | 2020 | Mexico City | Mexico | Incorporate a neurochemical imaging measure into risk calculator and assess its impact on prediction. | 19 | 1 (5.26) BIPS 18 (94.74%)APSS | 7 (36.84%) | SIPS | Referral | 20.33 non-converters 18.57 converters | 0.737 | Adding one clinical variable to a biological marker - each with substantial predictive properties - modestly improves the performance of either predictor alone and may yield a potentially useful imagingbased risk calculator for the prediction of conversion to syndromal psychosis | Overfitting, sample size, expensive method that limits its widespread use. | 4 |
| 26 | Brain functional connectivity data enhance prediction of clinical outcome in youth at risk for psychosis | Collin G^26^ | 2020 | Shanghai | China | Assesss a combined clinical and resting-state fMRI prediction model in adolescents and young adults at Clinical High Risk (CHR) | 137 | N/A | 36 (26.27%) poor outcome (including converters and treatment-refractory) | SIPS | Referral | 18.6 - good outcome 19.3 - intermediate outcome 18.6 - poor outcome | N/A | An imaging-only model yielded a significant prediction model, but a combined model including both clinical and connectivity measures showed the best performance. | Failed to reach a significant level od prediction, possible influences of head motion artifacts, post-hoc is not a proper confirmatory test | 5 |
| 27 | Childhood maltreatment in individuals at risk of psychosis: Results from the Brazilian SSAPP cohort | Freitas EL^27^ | 2020 | São Paulo | Brazil | Differences in childhood trauma between CHR x HC | 86 | N/A | NDA | SIPS | Outreach | 24.8 | 0.33 | Childhood trauma higher in CHR; physical abuse linked to hallucinations in CHR | Cross-sectional, assessment of childhood adversity | 5 |
| 28 | Clinical subtypes that predict conversion to psychosis: A canonical correlation analysis study from the ShangHai At Risk for Psychosis program | Zhang T^28^ | 2020 | Shanghai | China | Investigate whether subtypes defined by baseline clinical and cognitive features improve the prediction of psychosis | 289 | 7 (2.5%) BIPS 21 (7.4%) GRD | 54 (18.7%) | SOPS/ SIPS | Referral | 19 | 0.46 | The three different subtypes reflect significant differences in clinical and cognitive characteristics as well as in the risk of conversion to psychosis. | Single center sample recruitment, only 2 years follow-up, underestimation of the cllinical severity of their sample given the lost of participants with higher symptom, medication of CHR individuals, lack of other outcomes | 5 |
| 29 | Cognitive dysfunction in a psychotropic medication-naïve, clinical high-risk sample from the ShangHai-At-Risk-for-Psychosis (SHARP) study: Associations with clinical outcomes | Cui H^29^ | 2020 | Shanghai | China | Relate cognition to clinical outcome | 217 | N/A | 0.209 | SIPS | Referral | 18.6 | 0.47 | CHR showed small to medium deficits on cognition compared to HC; also between CHR-C and CHR-NC | sample not large enough; young sample (risk ahead); early reassessment (conversion ahead) | 5 |
| 30 | Conversion to psychosis in adolescents and adults: similar proportions, different predictors | Zhang TH^30^ | 2020 | Shangai | China | Investigate if adolescents differ from adults in conversion rates and predictors | 517 | 93% APSS, 11% BLIPS, 3% GRD | 107 (24.6%) | SIPS | Referral | 20.5 | 0.47 | Similar conversion rates. Negative symptoms predict conversion in adolescents, positive symptoms in adults | Single-center, psychotropic näive, GAF drop assessed retrospectively | 5 |
| 31 | Cortical Complexity in People at Ultra-High-Risk for Psychosis Moderated by Childhood Trauma | Hou J^31^ | 2020 | Shanghai | China | Measure cortical complexity in CHR | 36 | N/A | NDA | SIPS | Outreach | 19.1 | 0.333 | Moderating effect of childhood trauma in cortical folding, folding alterations in CHR | Small sample size, cross-sectional data | 5 |
| 32 | Functional connectome organization predicts conversion to psychosis in clinical high-risk youth from the SHARP program | Collin G^32^ | 2020 | Shanghai | China | Examine whether abnormalities in connectome organization precede psychosis onset | 158 | N/A | 23 (14.56%) | SIPS | Referral | 19.2 (converted) 18.7 (non-converted) | 0.506 | All CHRs, abnormal modular connectome organization at baseline was associated with a threefold conversion rate. A region-specific analysis showed that brain regions implicated in early-course schizophrenia and brain network organization precede the onset of psychosis. | Physiological and head motion artifact, biological validity of negative or anti-correlations, application of thresholds in functional network analyses, non-neural factors may have influenced the results | 5 |
| 33 | Higher number of minor physical anomalies correlates with frequency of prodromal symptoms in youth at elevated clinical risk for psychosis | Tikka DL^33^ | 2020 | Kanke | India | Assess the frequency of minor physical anomalies (MPAs) in youth clinically at-risk (CAR) for psychosis, compared to youth with first episode schizophrenia (FES), their unaffected first-degree relatives and healthy controls (HC) | 100 | N/A | NDA | PRIME | Referral + Outreach | 22.24 (CAR) 22.3 (FAR) | 0.6 | The ‘risk-marker’ status of minor physical anomalies (MPAs might) be extended beyond ‘familial at-risk’ onto ‘clinical at-risk’ as well | N/A | 4 |
| 34 | Lower prepulse inhibition in clinical high-risk groups but not in familial risk groups for psychosis compared with healthy controls | Togay B^34^ | 2020 | Istanbul | Turkey | Compare prespulse inhibition (PPI) in individuals with clinical and familial high risk for psychosis, and healthy controls | 29 | 32 APSS 1 BLIPS | NDA | CAARMS | Referral | 20.21 (CHR) 27.62 (FHR) | 0.759 | PPI was lower in the CHR group compared with both the FHR and control groups - and no difference in PPI between these two groups. | Help-seeking people, CAARMS criteria (may have excluded CHR with negative symptoms), small sample, lack of startle latency, the use of only 120 ms intervals | 4 |
| 35 | P300 as an index of transition to psychosis and of remission: Data from a clinical high risk for psychosis study and review of literature | Tang Y^35^ | 2020 | Shanghai | China | Address a question of both P300 oddball and novel abnormalities as predictors of transition to psychosis as well as predictors of remission from psychotic symptoms | 235 | N/A | ODDBALL: 19 (18.27%) NOVEL: 23 (17.56%) | PQ-b SIPS SOPS | Referral | ODDBALL: 19.7 (converted) 18.3 (non-converted)  NOVEL: 19.7 (converted) 18.6 (non-converted) | 0.5191 | P300 novel rather than P300 oddball was a better predictor of both conversion and remission. | N/A | 5 |
| 36 | Personality Traits as Markers of Psychosis Risk in Kenya: Assessment of Temperament and Character | Mamah D^36^ | 2020 | Nairobi | Kenya | Temperament and character in CHR | 268 | N/A | NDA | SIPS | Outreach | 21.1 | 0.526 | CHR have distinct personality traits | Applicability of TCI not established in Kenya | 5 |
| 37 | Poor functional recovery is better predicted than conversion in studies of outcomes of clinical high risk of psychosis: insight from SHARP | Zhang T^37^ | 2020 | Shanghai | China | Evaluate and compare the accuracy of predicting conversion v. poor functional outcome | 300 | N/A | 46 (23.1%) by NAPLS-2 risk calculator | SIPS | Referral | 19 | N/A | Risk calculator is better fit for predicting a poor functional outcome and tratment response than it is to predict conversion | Naturalistic cohort, effects of antipsychotic and other medication treatment, underestimation of the cllinical severity of their sample given the lost of participants with higher symptoms. | 5 |
| 38 | Real-world effectiveness of antipsychotic treatment in psychosis prevention in a 3-year cohort of 517 individuals at clinical high risk from the SHARP (ShangHai At Risk for Psychosis) | Zhang TH^38^ | 2020 | Shangai | China | Antipsychotics effect on rate of conversion to CHR | 450 | N/A | 108 (24%) | SIPS | Referral | 20.27 | 0.475 | Those without antipsychotics showed a lower conversion rate than those who took medication | Single site, innacuracy due to missing data or stigma, fucntional outcomes not assessed | 5 |
| 39 | Relationship Between Symptomatic Dimensions and Global Functioning of Non-Help-Seeking Individuals at Risk for Psychosis | Ayoub IA^39^ | 2020 | São Paulo | Brazil | Relationship between functioning and symptoms | 83 | N/A | NDA | SIPS | Outreach | 24.9 | 0.46 | Avolition was related to functioning | cross-sectional, use of GAF to assess functioning | 5 |
| 40 | Social cognitive endophenotypes in schizophrenia: A study comparing first episode schizophrenia patients and, individuals at clinical- and familial- 'at-risk' for psychosis | Tikka DL^40^ | 2020 | Kanke | India | Find out whether performance on social cognition tasks will distinguish ‘clinical at-risk (CAR) and ‘familial at-risk (FAR) individuals from remitted first episode schizophrenia (FES) patients and healthy controls | 100 | N/A | NDA | PRIME | Referral + Outreach | 22.24 (CAR) 22.3 (FAR) | 0.6 | Social cognitive measures (theory of mind and social perception) may be used as reliable endophenotype markers for schizophrenia and its sub-domains may be used for valid identification of AR individuals. | Lack of pronounced impairments in social functioning in at-risk groups, lack of prediction of psychosis data, limited to assessment of only emotion ‘identification’ and not emotion ‘discrimination’ or ‘differentiation’. | 4 |
| 41 | [Assessment of mental states at risk of psychotic transition in a sample of young male prisoners in Tunisia] | Fekih-Romdhane F^41^ | 2019 | Jendouba | Tunisia | Prevalence of CHR among newly incarcerated prisioners | 22 | N/A | NDA | CAARMS | Outreach | 28.7 | 1 | More social dysfunction, depression, anxiety, psychiatric family history, suicide attempts | small sample size, cross-sectional design, only males, self-report | 4 |
| 42 | Altered Cellular White Matter But Not Extracellular Free Water on Diffusion MRI in Individuals at Clinical High Risk for Psychosis | Tang Y^42^ | 2019 | Shanghai | China | Investigate whether or not cellular and extracellular alterations are already present in a predominantly medication-naive cohort of clinical high-risk individuals experiencing attenuated psychotic symptoms | 50 | 45 (90%) APSS 2 (4%) BIPS 1 (22%) GRDS 2 (4%) APSS + GRDS | 0.291 | SIPS | Referral | 19.7 | 0.6 | Cellular but not extracellular alterations characterized the clinical high-risk group, especially in those who experienced a decline in functioning | Cross-sectional nature, sample size, medication may contributed to the group differences | 5 |
| 43 | Altered resting-state functional connectivity of the insula in individuals with clinical high-risk and patients with first-episode schizophrenia | Li XB^43^ | 2019 | Beijing | China | determine to what degree abnormalities in insular functional connectivity occur in individuals with clinical high risk for psychosis (CHR), and whether which is associated with symptom severity | 24 | N/A | NDA | SIPS | Referral | 24.6 | 0.625 | Insular functional dysconnectivity with the sensory cortex may be a system-level neural substrate preceding the onset of psychosis. | Sample size, movement of head, possible failed to capture potentially important discrepancies across insular cortex given the first analysis from 3 subregions | 4 |
| 44 | Cognitive deficits in subjects at risk for psychosis, first-episode and chronic schizophrenia patients | Liu Y^44^ | 2019 | Shanghai | China | Use the MATRCIS battery test to investigate cognitive deficits in a large sample of subjects ranging from controls, SCZ, FEP, prodromal symptoms, subjects with genetic family risk (HRF) but no current symptoms and with prodromal symptoms (prodromal) | 73 | N/A | NDA | SIPS | Referral | 23.3 prodromal  25.6 High risk family | 0.581 | Prodromal subjects showed significant cognitive deficits which were similar in most domains to those found in patients with schizophrenia | SCZ patients under antipsychotic medication, did not considered socio-economic status or being raised in na urban vs rural environment | 5 |
| 45 | Functional asymmetry of thalamocortical networks in subjects at ultra-high risk for psychosis and first-episode schizophrenia | Zhu F^45^ | 2019 | Changsha | China | Determine the magnitude of functional asymmetry observed using the PAS method | 74 | N/A | NDA | SCID, SIPS, SOPS, PANSS | Referral | 22.04 (CHR) 23.3 (FEP) 21.37 (CONTROL) | 0.581 | CHR and FEP share decreased ‘Parameter of asymmetry’ (PAS) in the left thalamus, which can be used to dicriminate them from controls. | Sample size, lack of independent tests, low specificity of CHR classification | 5 |
| 46 | Gene expression over the course of schizophrenia: from clinical high-risk for psychosis to chronic stages | Ota VK^46^ | 2019 | São Paulo | Brazil | Find genes related to a prepsychotic stage,acute psychotic stage, long-term psychotic state, or following a long exposure to antipsychotics | 27 | N/A | CHR: 0 (0.0%) | CAARMS (CHR) DSM-IV SCID-I (FEP and SCZ) | Referral | 18.00 (CHR) 25.86 (FEP) 38.65 (SCZ) 33.51 (CONTROL) | 0.63 | Changes in gene expression profile throughout the different clinical stages of SCZ | Small sample, lack of folllow-up (longitudinal study), unmeasured potential confounder factors that might affect the results | 4 |
| 47 | Hearing spirits? Religiosity in individuals at risk for psychosis-Results from the Brazilian SSAPP cohort | Loch AA^47^ | 2019 | São Paulo | Brazil | Assess religiosity in CHR individuals | 79 | N/A | NDA | SIPS | Outreach | 24.6 | 0.329 | Organizational religious activity related to perceptual abnormalities | Small sample size | 5 |
| 48 | Higher order language impairments can predict the transition of ultrahigh risk state to psychosis-An empirical study | Obyedkov I^48^ | 2019 | Minsk | Belarus | Saccadic eye movement in SCZ x CHR x HC | 42 | N/A | NDA | CAARMS | Outreach | 21.8 | 1 | Saccadic abnormalities shown in SCZ and CHR in relation to HC | Lack of sample size calculation, confounding effect of antipsychotic use | 5 |
| 49 | Increased frontal gray matter volume in individuals with prodromal psychosis | Shan XX^49^ | 2019 | Changsha | China | Investigate anatomical deficits in prodromal individuals and their associations with clinical/cognitive features | 74 | 8 (10.81%) BIPS 44 (59.46%) APS 11 (14.86%) GRD 11 (14.86%) APS+GRD | NDA | SOPS/ SIPS | Referral | 22 | 0.5811 | Increased frontal gray matter volume in prodromal individuals | Cross-sectional study, lack of follow-up to verify transition and how the gray matter volume will be over time, lack of SCZ patients to compare, education is unmatched between two groups | 5 |
| 50 | Individuals at high risk for psychosis experience more childhood trauma, life events and social support deficit in comparison to healthy controls | Huang ZH^50^ | 2019 | Guangdong | China | Investigate childhood trauma, life events and social support in subjects with high risk for psychosis (HR), first episode psychosis with schizophrenia (FEP) and healthy control (HC). | 83 | N/A | NDA | SIPS | Referral | 28.8 | 0.53 | HR individuals experienced more childhood trauma, life events and social support deficit than HC group, which may be risk factors of conversion to psychosis | Lack of conversion rate, small sample size, use of retrospective scale (recall bias) | 5 |
| 51 | Network functional connectivity analysis in individuals at ultrahigh risk for psychosis and patients with schizophrenia | Chen X^51^ | 2019 | Beijing | China | Identify biomarkers for onset of schziphrenia based on a voxelwise whole-brain functional degree centrality (FDC) analysis | 30 | N/A | NDA | SIPS | Referral | 23.67 | 0.4667 | CHR subjects and patients with schizophrenia showed significantly increased FDC atthe medial prefrontal cortex (MPFC) and significantly decreased FDC at the right fusiform gyrus (FG) when compared with healthy controls | Lack of comparison to other mental disorders | 4 |
| 52 | Neurological soft signs and grey matter abnormalities in individuals with ultra-high risk for psychosis | Kong L^52^ | 2019 | Shanghai | China | Verify if neurological soft signs in CHR individuals would be accompanied by abnormal gre matter volume regions | 21 | N/A | NDA | SOPS/ SIPS | N/A | 17.81 | 0.5238 | there were significant associations between higher neurological soft signs values and reduced grey matter volume at the superior and medial frontal cortex, the pre- and post-central cortex, the insula, the caudate, and the cerebellum. | Small sample size, lack of matchd group | 4 |
| 53 | Occipital Alpha Connectivity During Resting-State Electroencephalography in Patients With Ultra-High Risk for Psychosis and Schizophrenia | Liu T^53^ | 2019 | Beijing | China | Investigate the resting-state functional connectivity of the alpha rhythm measured by electroencephalography (EEG) to reveal the relation between functional network and clinical symptoms | 21 | N/A | NDA | SIPS | N/A | 24.1 | 0.619 | Increased global efficiency, the local efficiency, and the path length were found in the FES and CHR groups compared with those of the HC group. | Limitation of EEG-based assessment for artefact removal | 4 |
| 54 | Olfactory and cognitive functions in Chinese individuals at clinical high risk for psychosis | He Y^54^ | 2019 | Changsha | China | Verify the theory that olfactory and cognitive functions are deficient in Chinese cohort with clinical high risk | 19 | N/A | NDA | SIPS | Referral | 20.47 | 0.7895 | The CHRs scored significantly lower than control int he olfactory identification test and all other cognitive tests, suggesting that olfactory dysfunction may be na early change of psychosis. | Small sample size, unbalenced gender ratio, lack of outcome. | 4 |
| 55 | Peripheral levels of superoxide dismutase and glutathione peroxidase in youths in ultra-high risk for psychosis: a pilot study | Zeni-Graiff M^55^ | 2019 | São Paulo | Brazil | Compare levels of superoxide dismutase and glutathione peroxidase in CHR vs. HC | 13 | N/A | NDA | CAARMS | Referral | 17.8 | 0.69 | Lower activity of selected enzimes in CHR compared to controls | small sample size, cross-sectional design | 4 |
| 56 | Prediction of psychosis in prodrome: development and validation of a simple, personalized risk calculator | Zhang T^56^ | 2019 | Shanghai | China | Derive and validate a risk calculator for predicting psychosis in CHR | 417 | 85.13% APSS 5.75% GRD 3.36% BIPS | 83 (19.9%) | SIPS | Referral | 20.9 | 0.48 | SIPS-RC calculator perform in same manner as NAPLS-2 | Limited number of CHRs in validation sample, lack of biomarkers in prediction, does not considered poor functioning as an outcome | 5 |
| 57 | Relationship between duration of untreated prodromal symptoms and symptomatic and functional recovery | Zhang T^57^ | 2019 | Shanghai | China | Analyse the association between the duration of untreated prodromal symptoms (DUPrS) and outcomes (symptomatic and functional recovery) in APS population | 391 | 100% APS | 82 (24.6%) | PQ-b SIPS SOPS | Referral | 20.4 (total) 20.2 (followed) | 0.47 | APS with longer DUPrS were correlated with poorer functional outcome, but not with poorer symptomatic outcome. DUPrS was related to poor functional outcome | Attrition and limited follow-up period, recall bias, did not consider the severity of negative symptoms, emotional or cognitive performances, individuals received naturalistic treatment | 5 |
| 58 | A comparative study of magnetic resonance imaging on the gray matter and resting-state function in prodromal and first-episode schizophrenia | Lian N^58^ | 2018 | Changsha | China | Investigate cerebral gray matter volume differences and resting-state functional connections among patients with psychosis risk syndrome (PRS), patients with first-episode schizophrenic (FES), and healthy controls (HC) | 19 | N/A | NDA | PRIME SIPS | Referral | 20.11 | 0.6842 | Gray matter volume of the right caudate nucleus was decreased in the patients with PRS and FES. Also, decreased gray matter volume in left fusiform gyrus and increased gray matter volume in bilateral putamen were found in PRS subjects, while in FES patients, there was a decrease in gray matter volume in the left caudate nucleus. | Small sample, PRS in early stages of schizophrenia which frontal lobe changes may not be apparrent, frontal lobe changes are not specifc to schziphrenia | 4 |
| 59 | A comparison of conversion rates, clinical profiles and predictors of outcomes in two independent samples of individuals at clinical high risk for psychosis in China | Li H^59^ | 2018 | Shanghai | China | Compare demographic and clinical characteristics of two samples (2011 and 2013) | 117 | 2011: 92 (78.6%) APSS 27 (23.1%) GRDS 4 (3.4%) BIPS  2013: 98 (98.0%) APSS 9 (9.0%) GRDS 3 (3.0%) BIPS | 2011: 25 (29.1%) 2013: 25 (27.5%) | SOPS/ SIPS | Referral | 2011: 24.7 2013: 21.0 | 0.43 | Conversion rates in 2013 sample are similar to those reported in non-Chinese samples and to the 2011 cohort. | N/A | 5 |
| 60 | Altered functional connectivity strength and its correlations with cognitive function in subjects with ultra-high risk for psychosis at rest | Li RR^60^ | 2018 | Changsha | China | Examine the altered patterns of functional connectivity strength (FCS) in whole-brain of CHR subjects | 34 | N/A | NDA | SOPS/ SIPS | Referral | 21.5 | 0.6176 | CHR subjects showed altered FCS in the frontal-occipital network. FCS values in the frontal-occipital network were significantly correlated to cognitive deficits in the CHR subjects. FCS values in the 2 abnormal brain regions was satisfactory able to discriminating the CHR subjects from the healthy controls | Sample size, lack of SCZ patients, need for longitudinal study, lack of follow up | 4 |
| 61 | Brain regional homogeneity and function connectivity in attenuated psychosis syndrome -based on a resting state fMRI study | Long X^61^ | 2018 | Shanghai | China | Explore brain functional alterations in Attenuated Psychosis Syndrome (APS) | 42 | 100% APSS | NDA | SIPS | Referral | 22.94 (after exclusion) | 0.4706 | APS subjects had spatially regional dysfunction and remoted synchronous dysfunction in the frontal and temporal lobes of the brain. Also, changes incombining regional homogeneity and functional connectivity patterns may reveal the mechanism of brain dysfunctions and may serve as an imaging biomarker for the diagnosis and evaluation of SCZ. | Limit samples, lack of follow-up data, templates used may not be fully applicable to Chinese population | 4 |
| 62 | Duration of untreated prodromal symptoms in a Chinese sample at a high risk for psychosis: demographic, clinical, and outcome | Zhang T^62^ | 2018 | Shanghai | China | Identify demographic and clinical factors contributing to the duration of untreated prodromal syndrome (DUPrS) and to evaluate the association with conversion to psychosis. | 391 | 100% APS 0% GRD (exclusion criteria) | 57 (14.57%) | SIPS | Referral | 20.4 | 0.473 | Either long or short DUPrS was not related to future psychosis onset. Individuals with APS were more likely to have a group of features associated with a longer DUPrS | Recall bias, confounders might play a role in the associations found, lack of direction from the influences.found | 5 |
| 63 | Identification and prediction of clinical high risk of psychosis in Chinese outpatients using two-stage screening | Xu L^63^ | 2018 | Shanghai | China |  | 112 | 98 (87.5%) APSS 6 (5.4%) BIPS 3 (2.7%) GRDS 5 (4.5%) APSS+GRDS | 27.5% CHR+ 1.7% CHR- | PS-R SIPS | Referral | 21.99 | 0.4107 | Two-stage screening indeed saves much time and alleviates the workload, but may exclude some target individuals. | Lack of investigation of selection bias, lack of questions about family history and general functions, lack of ditinguish individuals CHR from those with psychosis and psychotic symptoms, self-reported scale problems | 5 |
| 64 | Isolated hallucination is less predictive than thought disorder in psychosis: Insight from a longitudinal study in a clinical population at high risk for psychosis | Zhang T^64^ | 2018 | Shanghai | China | Evaluate the potential of frst-time experience of perceptual abnormalities (PAs) and/or thought content disorders (TCDs) to predict psychosis | 511 | N/A | 87 (19.64%) | SOPS/ SIPS | Referral | 20.6 | 0.472 | Higher conversion rate in CHRs with TCDs compared with those with PAs only. Compared with TCDs, the isolated PAs are not strongly associated with increased susceptibility to psychosis. | Observational study, various medications that the sample were taking, sample formed by outpatients of psychological clinic, age of eligible sample in current study was slightly biased against 14–21 years old | 5 |
| 65 | Relationship of obsessive-compulsive symptoms to clinical variables and cognitive functions in individuals at ultra high risk for psychosis | Soyata AZ^65^ | 2018 | Istanbul | Turkey | Evaluate the frequency of obsessive-compulsive symptoms (OCS) and their relationship with clinical variables and cognitive functions in individuals at CHR | 84 | 72% APS 17.9% BLIPS 10.1% family risk 8.33% APS + family risk 1.19% BLIPS + family risk | NDA | BPRS-E GAF | Referral | 20.6 | 0.702 | OCS are common in the CHR group and might be related to higher level of depression, but better work/study performance, and less cognitive deficits in this group | Help-seeking people, small sample, BPRS criteria (may have excluded CHR with negative symptoms), non blinded ratings for clinical scales (bias), did not use a scale of severity of obsessions and compulsions | 3 |
| 66 | Validating the Predictive Accuracy of the NAPLS-2 Psychosis Risk Calculator in a Clinical High-Risk Sample From the SHARP (Shanghai At Risk for Psychosis) Program | Zhang T^66^ | 2018 | Shanghai | China | Cross-validate the NAPLS-2 risk calculator in a Chinese clinical high-risk sample | 300 | N/A | 46 (23.1%) | SIPS | Referral | 19.1 | 0.472 | NAPLS-2 risk calculator has some generalizability to an Asian country and may have usefulness in clinical applications in China | N/A | 5 |
| 67 | Comorbid Mental Disorders and 6-Month Symptomatic and Functioning Outcomes in Chinese University Students at Clinical High Risk for Psychosis | Shi J^67^ | 2017 | Shanghai | China | Investigate the comorbid mental disorder in non-clinical CHR and the impact on attenuated psychosis symptoms (APS) as well as clinical outcome | 32 | 100% APSS 7 (21.875%) GRDS | 1 (3.7%) to psychosis 8 (61.5%) still CHR 4 (30.8%) full remission | SIPS | Outreach | 18.78 | 0.40625 | Half of the non-clinical CHR baseline found at least one non-psychotic comorbid mental disorder. The ones with non-psychotic comorbid mental disorders showed more serious APS as compared with those without comorbid mentaldisorders. | It is not possible to know if the comorbid mental disorders preceded or followed the development of CHR criteria, sample size, lack of data abotr the course of comorbid mental disorders | 5 |
| 68 | Poverty, low education, and the expression of psychotic-like experiences in the general population of São Paulo, Brazil | Loch AA^68^ | 2017 | São Paulo | Brazil | Assess psychotic-like experiences and sociodemographic correlated factors in the general population | 600 | N/A | NDA | PQ | Outreach | 24.1 | 0.499 | Poverty and low education might be associated with psychotic expression | Possible high proportion of false-positives, lack of information about help-seeking and stress, cross-sectional data, high refusal rate | 5 |
| 69 | Shorter leukocyte telomere length in patients at ultra high risk for psychosis | Maurya PK^69^ | 2017 | São Paulo | Brazil | Compare leukocyte telomere length (TL) between patients at CHR for psychosis and healthy controls (HC) | 22 | 33% TS 56% APS 11% BLIPS | NDA | CAARMS | Referral | 18.27 (CHR) 26.13 (CONTROL) | 0.636 | CHR had shorter telomare lenght than control | Small sample size, long time of storage before the analysis of CHR patients, lack of other measures | 4 |
| 70 | Systemic Therapy for Youth at Clinical High Risk for Psychosis: A Pilot Study | Shi J^70^ | 2017 | Shanghai | China | Evaluate the effect of systemic therapy (ST) for students at CHR on reducing symptoms and enhancing psychosocial function. | 26 | N/A | 1 (3.8%) | Chinese PQ-16 (CPQ-16)  SIPS | Outreach | 18.85 | 0.4615 | Systemic intervention for university students at CHR for psychosis may have a positive effect on symptoms and self-esteem as well as social support in short term | Sample size, standardized measures, time of follow-up assessments | 4 |
| 71 | Two-year follow-up of a Chinese sample at clinical high risk for psychosis: timeline of symptoms, help-seeking and conversion | Zhang TH^71^ | 2017 | Shanghai | China | examine baseline characteristics and the timing of symptom onset, help-seeking, or transition to psychosis over a 2-year period | 86 | N/A | 25 (21.4%) | SIPS | Referral | 24.7 | 0.479 | Converters had significantly longer delays in seeking help after first symptom | No control comparison group, Axis I diagnosis based on clinical judgement, GAF based on retrospective ratings, potential bias due to attrition | 5 |
| 72 | [The cellular factors of innate immunity in nonpsychotic patients at high risk for schizophrenia] | Vasilyeva EF^72^ | 2016 | Moscow | Russia | Find out whether the chanes in the parameters pf innate immunity take place prior to disease manifestation, and what role do they play in the pathogenesis of schizophrenia | 35 | non-psychotic mental disorders 13 mood disorder 14 personality disorder 8 schizotypal | NDA | Non-psychotic mental disorders SOPS CID-10 | Referral | 19.8 | 1 | Immune disturbances revealed in the study may play a role in the pathogenesis of the disease and have predictive value for schizophrenia | N/A | 4 |
| 73 | [The dynamics of psychopathological symptoms of ultra high risk for psychosis in young patients with non-psychotic mental disorders] | Omel'chenko MA^73^ | 2016 | Moscow | Russia | Describe the dynamics of psychopathological symptoms in young patients that met the criteria of ultra-high risk for chizophrenia who received preventive psychopharmacotherapy in 5-years follow-up | 32 | 7 (21.9%) mood disorders 12 (37.5%) personality disorder 13 (40.6%) schizotypal disorder | 1 (2.9%) manifest an attack of endogenous psychosis | Non-psychotic mental disorders of adolescence CID-10 | Referral | 16-25 years (mean not mentioned) | 1 | According to the characteristics of subsequent development of these disorders, four types were singled out: mechanisms of the development of acute sensitive delusions (I), interpretative delusions (II), catatonic disorganization (III) and cognitive disorders pathognomonic for schizophrenia spectrum disorders (IV ). Correlations between these types and nosologic disorders and their different reversibility under treatment were found. These types can be considered as predictors of outcome over the follow-up period. | N/A | 3 |
| 74 | Abnormal regional homogeneity as potential imaging biomarker for psychosis risk syndrome: a resting-state fMRI study and support vector machine analysis | Wang S^74^ | 2016 | Changsha | China | Compare regional homogeneity (ReHo) in 34 CHR vs. 37 HC | 34 | N/A | NDA | CAARMS + SIPS | Referral | 21.5 | 0.617 | Abnormal regional functional synchronization exists in CHR | Small sample size | 4 |
| 75 | Characterizing psychosis risk traits in Africa: A longitudinal study of Kenyan adolescents | Mamah D^75^ | 2016 | Machakos | Kenya | Investigate schizophrenia risk traits in Kenyan adolescents and identify predictors of psychosis progression. | 263 | N/A | 5 (3.8%) | WERCAP or SIPS | Outreach | 17.3 | 0.3965 | Psychopathology and disability occur at relatively high rates in Kenyan HR adolescent, where more psychiatric comorbidities,increased psychosocial stress, worse attention and better abstraction were observed in HR compared to LR partici-pants | Limited number of pschotic conversions, lack of alonger follow-up | 5 |
| 76 | Cognitive functioning in individuals at ultra-high risk for psychosis, first-degree relatives of patients with psychosis and patients with first-episode schizophrenia | Hou CL^76^ | 2016 | Guangdong | China | Compare cognitive functioning between CHRxHCxFEP | 40 | NDA | NDA | SIPS | Referral | 29.1 | 0.4 | Gradual decrease in cognitive functioning from HCm CHR and FEP groups | Cross-sectional, only familial risk, few cognitive domains studied, FEP not controlled for psychotropic drug use | 4 |
| 77 | Correlation of social cognition and neurocognition on psychotic outcome: a naturalistic follow-up study of subjects with attenuated psychosis syndrome | Zhang T^77^ | 2016 | Shanghai | China | Tested if APS increases dependence on neurocognition during the interpretation of others’ mental states / Theory of mind and neurocognition improves predictive accuracy of psychosis conversion | 83 | 100% APSS | 20 (25.6%) | SIPS | Referral | 19.1 | 0.578 | Mixed index 75% sensitivity and 69% accuracy. ToM and cognition were stronger related in CHR | short follow-up, observational follow-up, heterogeneous sample | 5 |
| 78 | Emotional Experiences Predict the Conversion of Individuals with Attenuated Psychosis Syndrome to Psychosis: A 6-Month Follow up Study | Chen FZ^78^ | 2016 | mainland China | China | Emotional experiences as predictors of conversion in CHR | 63 | 100% APS | 8(17%) | SIPS | Referral | 21.9 | 0.524 | Converters exhibited significantly poorer emotional experience and expression | Small sample size, short follow-up time, focus on symptoms and not in cognitive function | 4 |
| 79 | Faux pas recognition performance in a help-seeking population at clinical high risk of psychosis | Zhang T^79^ | 2016 | Shanghai | China | Impairment in theory of mind in CHR vs. Controls | 50 | N/A | 14 (28%) | SIPS | Referral | 20 | 0.52 | Baseline cognitive deficits for CHR vs controls, cognitive deficits CHR+ vs CHR- | No IQ test used, short follow-up period, only one cognitive sub-domain assessed | 5 |
| 80 | Patients with first-episode, drug-naive schizophrenia and subjects at ultra-high risk of psychosis shared increased cerebellar-default mode network connectivity at rest | Wang H^80^ | 2016 | Xiangya | China | Measure cerebellar-default mode network (DMN) connectivity in CHR, FEP and HC | 37 | N/A | NDA | SIPS | Referral | 21.5 | 0.617 | Increased cerebellar-default mode network connectivity in CHR and FEP | Small sample size | 4 |
| 81 | Peripheral immuno-inflammatory abnormalities in ultra-high risk of developing psychosis | Zeni-Graiff M^81^ | 2016 | São Paulo | Brazil | Compare cytokines in CHR x controls | 12 | N/A | NDA | CAARMS | Referral | 17 | 0.75 | Increased IL-6 and decreased IL-17 levels | Small sample size, cross-sectional, blood stored for too long | 4 |
| 82 | Protective factors in Chinese university students at clinical high risk for psychosis | Shi J^82^ | 2016 | Shanghai | China | Investigate if self-esteem, social support and resilience inluence CHR symptom severity | 32 | 100% APSS | 1(3.7%) | SIPS | Outreach | 18.8 | 0.406 | Lower self-esteem was the only determinant for negative, depressive symptoms and global functioning | Cross-sectional, protective factors assessed by self-report, university population | 5 |
| 83 | Reduced γ-Aminobutyric Acid and Glutamate+Glutamine Levels in Drug-Naïve Patients with First-Episode Schizophrenia but Not in Those at Ultrahigh Risk | Wang J^83^ | 2016 | Shanghai | China | Measure GABA, Glx in CHR, HC and FES by proton magnetic resonance spectroscopy | 21 | N/A | NDA | SIPS | Referral | 21.1 | 0.57 | GABA and Glx levels comparable in CHR and HC | RMI only collected in one region, small sample size | 4 |
| 84 | Theory of Mind Impairments in Youth at Clinical High Risk of Psychosis | Zhang TH^84^ | 2016 | Shangai | China | Investigate theory of mind in CHR | 40 | N/A | 7 (22.6%) | SIPS | Referral | 17.6 | 0.55 | Deficits in Theory of Mind capacity in CHR compared to HC, but scores are better than SCZ | No IQ test used, test not repeated at end of follow-up | 4 |
| 85 | Transition to Psychosis: Evaluation of the First-Degree Relatives of Patients with Schizophrenia ‎ | Hormozpour M^85^ | 2016 | Tehran | Iran | Conversion rate in first-degree relatives of patients with schizophrenia | 46 | 100% genetic risk | 6 (13%) | SIPS | Referral | 27.52 | 0.47 | Positive SIPS was a predictive factor for transition to psychosis in relatives of patients with schizophrenia | Small sample size, short duration of the follow-up, validity of some SIPS items for Persian speaking patients is in ‎question | 5 |
| 86 | [Information processing and brain metabolic characteristics in patients at ultra-high risk for endogenous psychosis] | Shendyapina MV^86^ | 2015 | Moscow | Russia | Study correlations between neurophysiological and neuroimaging parameters in ultrahigh risk patients | 56 | non-psychotic mental disorders 19 mood disorders 23 personality disorders 14 schizotypal disorders | N/A | Non-psychotic mental disorders SOPS CID-10 | Referral | N/A | 1 | Abnormality of sensory gating in patients at ultrahigh risk for endogenous psychosis that was not correlated with the metabolic parameters. | Sample of relatively young patients with potentially high neuronal plasticity, which could enhance the compensatory functions of the CNS. | 4 |
| 87 | Abnormalities in sleep patterns in individuals at risk for psychosis and bipolar disorder | Zanini MA^87^ | 2015 | São Paulo | Brazil | Compare sleep disturbances in CHR x controls | 20 | N/A | NDA | CAARMS | Referral | 18.3 | 0.65 | Worse sleep quality, increased sleep latency, increased rapid eye movement onset latency in ARMS | Small sample size, use of psychotropic medication | 4 |
| 88 | Cortico-Striatal GABAergic and Glutamatergic Dysregulations in Subjects at Ultra-High Risk for Psychosis Investigated with Proton Magnetic Resonance Spectroscopy | de la Fuente-Sandoval^88^ | 2015 | Mexico City | Mexico | Dysregulation in gamma-aminobuutyric acid and glutamete in CHR x HC | 23 | N/A | NDA | SIPS | Referral | 20.7 | 0.65 | Higher levels of gamma-aminobutiric acid and GLX in dorsal caudate and prefrontal cortex of CHR x HC | limited generazability of results, limitations inherent to neuroimaging technique | 4 |
| 89 | Family Perception and 6-Month Symptomatic and Functioning Outcomes in Young Adolescents at Clinical High Risk for Psychosis in a General Population in China | Wang L^89^ | 2015 | Shanghai | China | Investigate the characteristics and the role of family functioning in the development of CHR individuals among young adolescents | 32 | 100% APSS, 21% GRD | 0.125 | SIPS | Outreach | 18.8 | 0.594 | CHR families showed more maladaptive functioning | Large number of analysis (type I error), | 5 |
| 90 | Gene expression analysis in blood of ultra-high risk subjects compared to first-episode of psychosis patients and controls | Santoro ML^90^ | 2015 | São Paulo | Brazil | Investigate gene expression in CHR | 22 | 63% APSS, 18% BLIPS, 18% GRD | NDA | CAARMS | Referral | 18.3 | 0.63 | N/A | Small sample size, brief follow-up (<1 year) | 4 |
| 91 | History of childhood physical trauma is related to cognitive decline in individuals with ultra-high risk for psychosis | Üçok A^91^ | 2015 | Istanbul | Turkey | Investigate the relationship between childhood trauma (CT) and cognitive functioning in individuals with ultra-high risk for psychosis (CHR) | 53 | 72% APS 17.9% BLIPS 10.1% family risk 13.21% APS + family risk 1.86% BLIPS + family risk | NDA | BRPS | Referral | 21.1 | 0.736 | The findings suggest that a history of physical trauma has a negative impact on cognitive function in individuals at CHR for psychosis. | The results can not be generalized, help-seeking people, lack of information about other traumatic events, did not perfomed Bonferroni correction | 3 |
| 92 | Prodromal psychotic symptoms and psychological distress among secondary school students in Abeokuta, Nigeria | Okewole AO^92^ | 2015 | Abeokuta | Nigeria | Investigate the relationship between prodromal psychotic symptoms and psychological distress among Nigerian adolescents | 508 | NDA | NDA | PQ-B | Outreach | 15.4 | 0.37 | Relationship between reported prodromal symptoms and the occurrence of psychological distress, that early childhood trauma may be a predisposing factor to the early stages of development of psychosis, with female children being especially prone in the years of adolescence. | Lack of follow-up with SIPS | 5 |
| 93 | Psychosis risk screening among secondary school students in Abeokuta, Nigeria: Validity of the Prodromal Questionnaire - Brief Version (PQ-B) | Okewole AO^93^ | 2015 | Abeokuta | Nigeria | Assess the suitability of the Brief Version of the Prodromal Q0uestionnaire for use in an African population | 508 | NDA | NDA | PQ-B SIPS | Outreach | 15.4 | 0.37 | The strength of the PQ-B as evidenced by this study lies in its high negative predictive value. | Findings are not generalizable in Nigerian context, lack of family and other sources | 4 |
| 94 | Screening for schizophrenia in initial prodromal phase: Detecting the sub-threshold psychosis | Mohd Razali S^94^ | 2015 | Gelugor | Malaysia | Evaluate the efficacy of screening tool | 29 | NDA | NDA | CAARMS | Referral | N/A | N/A | Screening method (GHQ) is not suitable for screening early psychosis | Screening method (GHQ) is not suitable for screening early psychosis | 4 |
| 95 | Screening schizotypal personality disorder for detection of clinical high risk of psychosis in Chinese mental health services | Zhang T^95^ | 2015 | Shanghai | China | Schizotypal personality disorder screening questionnaire helps CHR detection | 91 | N/A | NDA | SIPS | Referral | 25.9 | 0.505 | Identifying SPD may be useful in detecting CHR | cross-sectional, only included Chinese clinical population, Axis I diagnosis obtained from medical records | 5 |
| 96 | Spectral editing in proton magnetic resonance spectroscopy. Determination of GABA level in the brains of humans with ultra-high risk for schizophrenia | Men'shchikov PE^96^ | 2015 | Moscow | Russia | Measure imbalance of inhibitory and excitatory neurotransmitters in the brain frontal lobes | 21 | N/A | NDA | SIPS | Referral | 20.3 | 1 | Decrease in GABA and GABA/GLX in CHR lef brain hemisphere | N/A | 4 |
| 97 | Identification and characterization of college students with attenuated psychosis syndrome in China | Chen F^97^ | 2014 | Shanghai | China | Test whether APS could be detected in a college population by self-report | 20 | 100% APS | NDA | SIPS | Outreach | 18.9 | 0.55 | APS can be detected in college sample, psychosis risk symptoms are associated with co-ocurring psychopathology | Recruitment from mental health education course, two-stage screening process, co-occurring psychopathology assessed with self-report instrument | 5 |
| 98 | Mismatch Negativity reduction in the left cortical regions in first-episode psychosis and in individuals at ultra high-risk for psychosis | Solís-Vivanco R^98^ | 2014 | Mexico City | Mexico | Explore the scalp distribution of MMN in FEP patients, CHR subjects and healthy controls. | 20 | N/A | NDA | SIPS | Referral | 20.8 | 0.65 | Amplitude reduction of duration MMN in FEP patients and CHR individuals,especially in the left cortical regions | Small sample, cross-sectional study limitations | 4 |
| 99 | Personality features in ultra-high risk for psychosis: a comparative study with schizophrenia and control subjects using the Temperament and Character Inventory-Revised (TCI-R) | Fresán A^99^ | 2014 | Mexico City | Mexico | Compare temperament and character dimensions between CHR individuals, patients with schizophreniaand healthy controls | 25 | N/A | NDA | SIPS | Referral | 19.56 | 0.76 | Higher levels of Harm Avoidance are present both in CHR individuals and patients with schizophrenia, while lower scores than controls in respec to Cooperativeness | Cross-sectional design | 4 |
| 100 | Prolonged cortical silent period among drug-naive subjects at ultra-high risk of psychosis | Tang Y^100^ | 2014 | Shanghai | China | Compare cortical inhibition CHRxHCxSCZ | 16 | 100% APSS | NDA | SIPS | Referral | 20.9 | 0.562 | SCZ and CHR have prolonged cortical silent period compared to HC | small sample size, cross-sectional design | 4 |
| 101 | Validation of the Arabic version of the Comprehensive Assessment of At Risk Mental States (CAARMS) in Tunisian adolescents and young adults | Braham A^101^ | 2014 | Sousse | Tunisia | Examine whether the Arabic version of the CAARMS has good construct validity, concurrent validity and reliability | 22 | N/A | NDA | CAARMS | Referral | 20 | 0.5454 | CAARMS version translated into Arabic is valid and reliable. | Small sample size | 4 |
| 102 | Cognitive deficits in clinical and familial high risk groups for psychosis are common as in first episode schizophrenia | Üçok A^102^ | 2013 | Istanbul | Turkey | Compare the neurocognitive functions in individuals with clinical or genetic risk for psychosis, in patients with FES and in healthy controls. | 52 | 18.8% BLIPS 69.6% APSS 11.6% family risk 13.46% APS + family risk 3.85% BLIPS + family risk | NDA | BPRS Family history and GAF | Referral | 21.2 | 0.692 | No difference in cognitive performances of CHR and FHR, and cognitive deficits in CHR and FHR groups were largely similar to those with FES | Referral, small sample size, CHR criteria, cross-sectional | 4 |
| 103 | Reduced P3a amplitudes in antipsychotic naïve first-episode psychosis patients and individuals at clinical high-risk for psychosis | Mondragón-Maya A^103^ | 2013 | Mexico City | Mexico | Analyze the MMN-P3a complex in first-episode, at clinical high-risk for psychosis and healthy controls | 23 | N/A | NDA | SIPS | Referral | 20.1 | 0.6956 | Although a P3a decrement in chronic schizophrenia and FEP has been previously reported, our results suggest that this novelty detection impairment is present even in pre-psychosis stages | Small sample, lack of cognitive evaluations or sctrutural MRI analyses, cross-sectional study' | 4 |
| 104 | Striatal glutamate and the conversion to psychosis: a prospective 1H-MRS imaging study | de la Fuente-Sandoval^104^ | 2013 | Mexico City | Mexico | See if increased glutamate levels in associative-striatum predicts psychosis in CHR | 19 | N/A | 7 (37%) | SIPS | Referral | 19.7 | 0.737 | Transition group had higher glutamate levels compared to non-transition and controls | Small sample size | 4 |
| 105 | The history of childhood trauma among individuals with ultra high risk for psychosis is as common as among patients with first-episode schizophrenia | Sahin S^105^ | 2013 | Istanbul | Turkey | Investigate the severity of childhood trauma and its relationship with clinical features in first-episode schizophrenia and ultrahigh risk for psychosis | 41 | N/A | NDA | BPRS Family history and GAF | Referral | 20.5 | 0.6829 | Emotional and physicalabuse, physical and emotionalneglect scores and childhood trauma (CT) of both CHR group and FES were higher than the control. CT is related to the severity of psychoticsymptoms in both groups. | Referral, limitation given the CHR definition, used self-reported scales, CHR small sample size, cross-sectional study design | 4 |
| 106 | A survey of psychosis risk symptoms in Kenya | Mamah D^106^ | 2012 | Nairobi | Kenya | Measure the prevalence of psychosis risk in a community sample in Nairobi, Kenya, and to evaluate the effects of key demographic variables. | 408 | 101 (11.1%) high-symptom 169 (18.6%) intermediate-symptom 138 (15.2%) grandiose-symptom | NDA | mPRIME | Outreach | 20.4 high-symptom 18.8 intermediate-symptom 18.1 grandiose-symptom | 0.607 | Psychosis risk symptoms appear to be highly prevalent in Kenyan youth: 45.5% reported having had any psychosis risk symptom, higher scores in females of persecutory ideation and auditory hallucinations. | mPRIME may overestimate the risk, self-report study, questionnaire items were not fully completed by all participant, cross-sectional | 4 |
| 107 | Higher Levels of Glutamate in the Associative-Striatum of Subjects with Prodromal Symptoms of Schizophrenia and Patients with First-Episode Psychosis | de la Fuente-Sandoval^107^ | 2011 | Mexico City | Mexico | Compare glutamate levels in the precomissural dorsal-caudate and cerebellar cortex in CHR, FEP, HC | 18 | N/A | NDA | SIPS | Referral | 19.56 | 0.777 | CHR and FEP showed higher levels of glutamate | No cognitive tests, not matched for education, limitations specific to the neuroimaging protocol used | 4 |
| 108 | Neurocognitive assessment of ultra high risk of psychosis states using the MATRICS battery (Measurement and Treatment Research to Improve Cognition in Schizophrenia) | Serrani D^108^ | 2010 | Rosário | Argentina | Asses neuropsychological profile of subjects at CHR for psychosis | 27 | 63% APSS, 11% BLIPS, 26% GRD | NDA | CAARMS | Referral | 17.4 | 0.81 | CHR scored below in working memory, verbal and visual memory, and social cognition | Small sample, difficulty imposed by using IQ as a group comparison factor, lack of standardized application norms for MATRICS in other clinical populations besides schizophrenics, absence of validation studies in argentine population | 4 |
| 109 | Altered Resting State Functional Connectivity and Its Correlation with Cognitive Functions at Ultra High Risk for Psychosis | Bulbul O^109^ | 2022 | Istambul | China | Identify resting state functional connectivity alterations and correlations with CHR neuropsychological tests | 20 | N/A | NDA | CAARMS | Referral | 20,3 | 0,20 | Connectivity was related to trail making test scores in CHR | Small sample size, recruitment from tertiary sector | 2 |
| 110 | Relationship Between Cognitive and Clinical Insight at Different Durations of Untreated Attentional Psychotic Symptoms in High-Risk Individuals | Xu L^110^ | 2021 | Shanghai | China | Wether cognitive insight is impaired in CHR | 121 | 100% APS | NDA | SIPS | Referral | 18,37 | 0,45 | APS have impaired cognitive insight, lower self-reflectiveness | Data of HC collected by teachers, sample size | 3 |
| 111 | MRI-Based Markers of Changes in the Supragranular Cortical Layer in Individuals at Clinically High Risk of Endogenous Psychosis | Tomyshev AS^111^ | 2021 | Moscow | China | Search for abnormalities in cortical layers | 33 | N/A | NDA | SIPS | Referral | 20,1 | 1,00 | Predominance of supragranular thinning in comparison with a decrease of infragranular cortical layer thickness | N/A | 3 |
| 112 | Progressive decline of cognition during the conversion from prodrome to psychosis with a characteristic pattern of the theory of mind compensated by neurocognition | Zhang T^112^ | 2018 | Shanghai | China | Explore neurocognitive performances in CHR | 83 | 100% APS | 26, 31% | SIPS | Referral | 19,4 | 0,57 | Path from neurocognition to ToM was statistically significant for CHR | Cognitive battery only at baseline, no comprehensive ToM measure | 2 |

**Abbreviations:**

CHR: clinical high-risk

FEP: First episode psychosis

HC: healthy control

SCZ: schizophrenia

APSS: attenuated psychotic symptoms syndrome

BLIPS: brief intermittent psychotic symptoms

GRD: genetic risk and functional decline syndrome

SIPS: Structured Interview for Prodromal Syndromes

CAARMS: Comprehensive Assessment for At Risk Mental States

N/A: not available

NDA: not applicable

**References**

1. Haddad, Natalia Mansur *et al.* Cognitive patterns and conversion in a representative sample of individuals at risk for psychosis. *Journal of Nervous and Mental Diseases* **(in press)**, (2021).

2. Mamah, D., Mutiso, V. N. & Ndetei, D. M. Longitudinal and cross-sectional validation of the WERCAP screen for assessing psychosis risk and conversion. *Schizophr Res* **241**, 201–209 (2022).

3. Zhang, T. *et al.* Neurocognitive Assessments Are More Important Among Adolescents Than Adults for Predicting Psychosis in Clinical High Risk. *Biol Psychiatry Cogn Neurosci Neuroimaging* **7**, 56–65 (2022).

4. Li, Z. *et al.* Plasma metabolic alterations and potential biomarkers in individuals at clinical high risk for psychosis. *Schizophr Res* **239**, 19–28 (2022).

5. Joaquim, H. P. G. *et al.* Plasmatic endocannabinoids are decreased in subjects with ultra-high risk of psychosis. *Eur J Neurosci* (2021) doi:10.1111/ejn.15509.

6. Del Re, E. C. *et al.* Baseline Cortical Thickness Reductions in Clinical High Risk for Psychosis: Brain Regions Associated with Conversion to Psychosis Versus Non-Conversion as Assessed at One-Year Follow-Up in the Shanghai-At-Risk-for-Psychosis (SHARP) Study. *Schizophr Bull* **47**, 562–574 (2021).

7. Zhang, T. *et al.* Calculating individualized risk components using a mobile app-based risk calculator for clinical high risk of psychosis: findings from ShangHai At Risk for Psychosis (SHARP) program. *Psychol Med* **51**, 653–660 (2021).

8. Luo, X. *et al.* Changes in the cognitive function of Chinese college students with a clinical high risk of psychosis. *Psychiatry Res* **305**, 114242 (2021).

9. Pereira, C. A. C. *et al.* COX-2 pathway is upregulated in ultra-high risk individuals for psychosis. *World J Biol Psychiatry* 1–6 (2021) doi:10.1080/15622975.2021.1961501.

10. Li, H. *et al.* Enhancing attention and memory of individuals at clinical high risk for psychosis with mHealth technology. *Asian J Psychiatr* **58**, 102587 (2021).

11. Ventura, J. *et al.* Establishing a clinical high-risk program in Tunisia, North Africa: A pilot study in early detection and identification. *Early Interv Psychiatry* **15**, 1777–1783 (2021).

12. Zhang, T. *et al.* Further evidence that antipsychotic medication does not prevent long-term psychosis in higher-risk individuals. *Eur Arch Psychiatry Clin Neurosci* **272**, 591–602 (2022).

13. Bo, Q. *et al.* Impaired Sensorimotor Gating Using the Acoustic Prepulse Inhibition Paradigm in Individuals at a Clinical High Risk for Psychosis. *Schizophr Bull* **47**, 128–137 (2021).

14. Talib, L. L. *et al.* Increased PLA2 activity in individuals at ultra-high risk for psychosis. *Eur Arch Psychiatry Clin Neurosci* **271**, 1593–1599 (2021).

15. He, X.-Y. *et al.* Individuals at ultra-high risk of psychosis and first-degree relatives of patients with schizophreniaexperience impaired family functionality and social support deficit in comparison to healthy controls. *Compr Psychiatry* **109**, 152263 (2021).

16. Nogueira, A. S. *et al.* Influence of migration on the thought process of individuals at ultra-high risk for psychosis. *Braz J Psychiatry* **43**, 285–288 (2021).

17. Mamah, D., Mutiso, V. N. & Ndetei, D. M. Neurocognition in Kenyan youth at clinical high risk for psychosis. *Schizophr Res Cogn* **25**, 100198 (2021).

18. Üçok, A. *et al.* Relationship of negative symptom severity with cognitive symptoms and functioning in subjects at ultra-high risk for psychosis. *Early Interv Psychiatry* **15**, 966–974 (2021).

19. Cui, G. *et al.* Salivary Metabolomics Reveals that Metabolic Alterations Precede the Onset of Schizophrenia. *J Proteome Res* **20**, 5010–5023 (2021).

20. Wu, J. *et al.* Screening of the college students at clinical high risk for psychosis in China: a multicenter epidemiological study. *BMC Psychiatry* **21**, 253 (2021).

21. Zhang, T. *et al.* Subtypes of Clinical High Risk for Psychosis that Predict Antipsychotic Effectiveness in Long-Term Remission. *Pharmacopsychiatry* **54**, 23–30 (2021).

22. Wu, G. *et al.* Temporal and time-frequency features of auditory oddball response in distinct subtypes of patients at clinical high risk for psychosis. *Eur Arch Psychiatry Clin Neurosci* **272**, 449–459 (2022).

23. Ren, Y. *et al.* A Weighted Gene Co-expression Network Analysis Reveals lncRNA Abnormalities in the Peripheral Blood Associated With Ultra-High-Risk for Psychosis. *Front Psychiatry* **11**, 580307 (2020).

24. Pu, C. *et al.* Altered cerebellocerebral structural covariance in individuals with attenuated psychosis syndrome. *Asian J Psychiatr* **53**, 102238 (2020).

25. Kegeles, L. S. *et al.* An imaging-based risk calculator for prediction of conversion to psychosis in clinical high-risk individuals using glutamate 1H MRS. *Schizophr Res* **226**, 70–73 (2020).

26. Collin, G. *et al.* Brain functional connectivity data enhance prediction of clinical outcome in youth at risk for psychosis. *Neuroimage Clin* **26**, 102108 (2020).

27. Freitas, E. L. *et al.* Childhood maltreatment in individuals at risk of psychosis: Results from the Brazilian SSAPP cohort. *Int J Soc Psychiatry* **66**, 566–575 (2020).

28. Zhang, T. *et al.* Clinical subtypes that predict conversion to psychosis: A canonical correlation analysis study from the ShangHai At Risk for Psychosis program. *Aust N Z J Psychiatry* **54**, 482–495 (2020).

29. Cui, H. *et al.* Cognitive dysfunction in a psychotropic medication-naïve, clinical high-risk sample from the ShangHai-At-Risk-for-Psychosis (SHARP) study: Associations with clinical outcomes. *Schizophr Res* **226**, 138–146 (2020).

30. Zhang, T. *et al.* Conversion to psychosis in adolescents and adults: similar proportions, different predictors. *Psychol Med* 1–9 (2020) doi:10.1017/S0033291720000756.

31. Hou, J. *et al.* Cortical Complexity in People at Ultra-High-Risk for Psychosis Moderated by Childhood Trauma. *Front Psychiatry* **11**, 594466 (2020).

32. Collin, G. *et al.* Functional connectome organization predicts conversion to psychosis in clinical high-risk youth from the SHARP program. *Mol Psychiatry* **25**, 2431–2440 (2020).

33. Tikka, D. L., Singh, A. R. & Tikka, S. K. Higher number of minor physical anomalies correlates with frequency of prodromal symptoms in youth at elevated clinical risk for psychosis. *Asian J Psychiatr* **47**, 101869 (2020).

34. Togay, B. *et al.* Lower prepulse inhibition in clinical high-risk groups but not in familial risk groups for psychosis compared with healthy controls. *Early Interv Psychiatry* **14**, 196–202 (2020).

35. Tang, Y. *et al.* P300 as an index of transition to psychosis and of remission: Data from a clinical high risk for psychosis study and review of literature. *Schizophr Res* **226**, 74–83 (2020).

36. Mamah, D. *et al.* Personality Traits as Markers of Psychosis Risk in Kenya: Assessment of Temperament and Character. *Schizophr Bull Open* **1**, sgaa051 (2020).

37. Zhang, T. *et al.* Poor functional recovery is better predicted than conversion in studies of outcomes of clinical high risk of psychosis: insight from SHARP. *Psychol Med* **50**, 1578–1584 (2020).

38. Zhang, T. *et al.* Real-world effectiveness of antipsychotic treatment in psychosis prevention in a 3-year cohort of 517 individuals at clinical high risk from the SHARP (ShangHai At Risk for Psychosis). *Aust N Z J Psychiatry* **54**, 696–706 (2020).

39. Ayoub, I. A. *et al.* Relationship Between Symptomatic Dimensions and Global Functioning of Non-Help-Seeking Individuals at Risk for Psychosis. *J Nerv Ment Dis* **208**, 953–957 (2020).

40. Tikka, D. L., Singh, A. R. & Tikka, S. K. Social cognitive endophenotypes in schizophrenia: A study comparing first episode schizophrenia patients and, individuals at clinical- and familial- ‘at-risk’ for psychosis. *Schizophr Res* **215**, 157–166 (2020).

41. Fekih-Romdhane, F., Labidi, A., Ridha, R. & Cheour, M. [Assessment of mental states at risk of psychotic transition in a sample of young male prisoners in Tunisia]. *Encephale* **46**, 348–355 (2020).

42. Tang, Y. *et al.* Altered Cellular White Matter But Not Extracellular Free Water on Diffusion MRI in Individuals at Clinical High Risk for Psychosis. *Am J Psychiatry* **176**, 820–828 (2019).

43. Li, X.-B. *et al.* Altered resting-state functional connectivity of the insula in individuals with clinical high-risk and patients with first-episode schizophrenia. *Psychiatry Res* **282**, 112608 (2019).

44. Liu, Y. *et al.* Cognitive deficits in subjects at risk for psychosis, first-episode and chronic schizophrenia patients. *Psychiatry Res* **274**, 235–242 (2019).

45. Zhu, F. *et al.* Functional asymmetry of thalamocortical networks in subjects at ultra-high risk for psychosis and first-episode schizophrenia. *Eur Neuropsychopharmacol* **29**, 519–528 (2019).

46. Ota, V. K. *et al.* Gene expression over the course of schizophrenia: from clinical high-risk for psychosis to chronic stages. *NPJ Schizophr* **5**, 5 (2019).

47. Loch, A. A. *et al.* Hearing spirits? Religiosity in individuals at risk for psychosis-Results from the Brazilian SSAPP cohort. *Schizophr Res* **204**, 353–359 (2019).

48. Pawełczyk, A., Łojek, E., Żurner, N., Kotlicka-Antczak, M. & Pawełczyk, T. Higher order language impairments can predict the transition of ultrahigh risk state to psychosis-An empirical study. *Early Interv Psychiatry* **15**, 314–327 (2021).

49. Shan, X.-X. *et al.* Increased frontal gray matter volume in individuals with prodromal psychosis. *CNS Neurosci Ther* **25**, 987–994 (2019).

50. Huang, Z.-H. *et al.* Individuals at high risk for psychosis experience more childhood trauma, life events and social support deficit in comparison to healthy controls. *Psychiatry Res* **273**, 296–302 (2019).

51. Chen, X. *et al.* Network functional connectivity analysis in individuals at ultrahigh risk for psychosis and patients with schizophrenia. *Psychiatry Res Neuroimaging* **290**, 51–57 (2019).

52. Kong, L. *et al.* Neurological soft signs and grey matter abnormalities in individuals with ultra-high risk for psychosis. *Psych J* **8**, 252–260 (2019).

53. Liu, T. *et al.* Occipital Alpha Connectivity During Resting-State Electroencephalography in Patients With Ultra-High Risk for Psychosis and Schizophrenia. *Front Psychiatry* **10**, 553 (2019).

54. He, Y. *et al.* Olfactory and cognitive functions in Chinese individuals at clinical high risk for psychosis. *Psychiatry Res* **272**, 51–53 (2019).

55. Zeni-Graiff, M. *et al.* Peripheral levels of superoxide dismutase and glutathione peroxidase in youths in ultra-high risk for psychosis: a pilot study. *CNS Spectr* **24**, 333–337 (2019).

56. Zhang, T. *et al.* Prediction of psychosis in prodrome: development and validation of a simple, personalized risk calculator. *Psychological Medicine* **49**, 1990–1998 (2019).

57. Zhang, T. *et al.* Relationship between duration of untreated prodromal symptoms and symptomatic and functional recovery. *Eur Arch Psychiatry Clin Neurosci* **269**, 871–877 (2019).

58. Lian, N. *et al.* A comparative study of magnetic resonance imaging on the gray matter and resting-state function in prodromal and first-episode schizophrenia. *Am J Med Genet B Neuropsychiatr Genet* **177**, 537–545 (2018).

59. Li, H. *et al.* A comparison of conversion rates, clinical profiles and predictors of outcomes in two independent samples of individuals at clinical high risk for psychosis in China. *Schizophr Res* **197**, 509–515 (2018).

60. Li, R.-R. *et al.* Altered functional connectivity strength and its correlations with cognitive function in subjects with ultra-high risk for psychosis at rest. *CNS Neurosci Ther* **24**, 1140–1148 (2018).

61. Long, X. *et al.* Brain regional homogeneity and function connectivity in attenuated psychosis syndrome -based on a resting state fMRI study. *BMC Psychiatry* **18**, 383 (2018).

62. Zhang, T. *et al.* Duration of untreated prodromal symptoms in a Chinese sample at a high risk for psychosis: demographic, clinical, and outcome. *Psychol Med* **48**, 1274–1281 (2018).

63. Xu, L. *et al.* Identification and prediction of clinical high risk of psychosis in Chinese outpatients using two-stage screening. *Schizophr Res* **202**, 284–290 (2018).

64. Zhang, T. *et al.* Isolated hallucination is less predictive than thought disorder in psychosis: Insight from a longitudinal study in a clinical population at high risk for psychosis. *Sci Rep* **8**, 13962 (2018).

65. Soyata, A. Z., Akışık, S., İnhanlı, D., Noyan, H. & Üçok, A. Relationship of obsessive-compulsive symptoms to clinical variables and cognitive functions in individuals at ultra high risk for psychosis. *Psychiatry Res* **261**, 332–337 (2018).

66. Zhang, T. *et al.* Validating the Predictive Accuracy of the NAPLS-2 Psychosis Risk Calculator in a Clinical High-Risk Sample From the SHARP (Shanghai At Risk for Psychosis) Program. *Am J Psychiatry* **175**, 906–908 (2018).

67. Shi, J. *et al.* Comorbid Mental Disorders and 6-Month Symptomatic and Functioning Outcomes in Chinese University Students at Clinical High Risk for Psychosis. *Front Psychiatry* **8**, 209 (2017).

68. Loch, A. A. *et al.* Poverty, low education, and the expression of psychotic-like experiences in the general population of São Paulo, Brazil. *Psychiatry Res* **253**, 182–188 (2017).

69. Maurya, P. K. *et al.* Shorter leukocyte telomere length in patients at ultra high risk for psychosis. *Eur Neuropsychopharmacol* **27**, 538–542 (2017).

70. Shi, J. *et al.* Systemic Therapy for Youth at Clinical High Risk for Psychosis: A Pilot Study. *Front Psychiatry* **8**, 211 (2017).

71. Zhang, T. H. *et al.* Two-year follow-up of a Chinese sample at clinical high risk for psychosis: timeline of symptoms, help-seeking and conversion. *Epidemiol Psychiatr Sci* **26**, 287–298 (2017).

72. Vasilyeva, E. F. *et al.* [The cellular factors of innate immunity in nonpsychotic patients at high risk for schizophrenia]. *Zh Nevrol Psikhiatr Im S S Korsakova* **116**, 60–65 (2016).

73. Omel’chenko, M. A., Rumyantsev, A. O. & Kaleda, V. G. [The dynamics of psychopathological symptoms of ultra high risk for psychosis in young patients with non-psychotic mental disorders]. *Zh Nevrol Psikhiatr Im S S Korsakova* **116**, 16–21 (2016).

74. Wang, S. *et al.* Abnormal regional homogeneity as potential imaging biomarker for psychosis risk syndrome: a resting-state fMRI study and support vector machine analysis. *Sci Rep* **6**, 27619 (2016).

75. Mamah, D. *et al.* Characterizing psychosis risk traits in Africa: A longitudinal study of Kenyan adolescents. *Schizophr Res* **176**, 340–348 (2016).

76. Hou, C.-L. *et al.* Cognitive functioning in individuals at ultra-high risk for psychosis, first-degree relatives of patients with psychosis and patients with first-episode schizophrenia. *Schizophr Res* **174**, 71–76 (2016).

77. Zhang, T. *et al.* Correlation of social cognition and neurocognition on psychotic outcome: a naturalistic follow-up study of subjects with attenuated psychosis syndrome. *Sci Rep* **6**, 35017 (2016).

78. Chen, F. Z. *et al.* Emotional Experiences Predict the Conversion of Individuals with Attenuated Psychosis Syndrome to Psychosis: A 6-Month Follow up Study. *Front Psychol* **7**, 818 (2016).

79. Zhang, T. *et al.* Faux pas recognition performance in a help-seeking population at clinical high risk of psychosis. *Eur Arch Psychiatry Clin Neurosci* **266**, 71–78 (2016).

80. Wang, H. *et al.* Patients with first-episode, drug-naive schizophrenia and subjects at ultra-high risk of psychosis shared increased cerebellar-default mode network connectivity at rest. *Sci Rep* **6**, 26124 (2016).

81. Zeni-Graiff, M. *et al.* Peripheral immuno-inflammatory abnormalities in ultra-high risk of developing psychosis. *Schizophr Res* **176**, 191–195 (2016).

82. Shi, J. *et al.* Protective factors in Chinese university students at clinical high risk for psychosis. *Psychiatry Res* **239**, 239–244 (2016).

83. Wang, J. *et al.* Reduced γ-Aminobutyric Acid and Glutamate+Glutamine Levels in Drug-Naïve Patients with First-Episode Schizophrenia but Not in Those at Ultrahigh Risk. *Neural Plast* **2016**, 3915703 (2016).

84. Zhang, T. *et al.* Theory of Mind Impairments in Youth at Clinical High Risk of Psychosis. *Psychiatry* **79**, 40–55 (2016).

85. Hormozpour, M. *et al.* Transition to Psychosis: Evaluation of the First-Degree Relatives of Patients with Schizophrenia ‎. *Iran J Psychiatry* **11**, 15–23 (2016).

86. Shendyapina, M. V. *et al.* [Information processing and brain metabolic characteristics in patients at ultra-high risk for endogenous psychosis]. *Zh Nevrol Psikhiatr Im S S Korsakova* **115**, 24–29 (2015).

87. Zanini, M. A. *et al.* Abnormalities in sleep patterns in individuals at risk for psychosis and bipolar disorder. *Schizophr Res* **169**, 262–267 (2015).

88. de la Fuente-Sandoval, C. *et al.* Cortico-Striatal GABAergic and Glutamatergic Dysregulations in Subjects at Ultra-High Risk for Psychosis Investigated with Proton Magnetic Resonance Spectroscopy. *Int J Neuropsychopharmacol* **19**, pyv105 (2015).

89. Wang, L. *et al.* Family Perception and 6-Month Symptomatic and Functioning Outcomes in Young Adolescents at Clinical High Risk for Psychosis in a General Population in China. *PLoS One* **10**, e0138361 (2015).

90. Santoro, M. L. *et al.* Gene expression analysis in blood of ultra-high risk subjects compared to first-episode of psychosis patients and controls. *World J Biol Psychiatry* **16**, 441–446 (2015).

91. Üçok, A. *et al.* History of childhood physical trauma is related to cognitive decline in individuals with ultra-high risk for psychosis. *Schizophr Res* **169**, 199–203 (2015).

92. Okewole, A. O. *et al.* Prodromal psychotic symptoms and psychological distress among secondary school students in Abeokuta, Nigeria. *J Child Adolesc Ment Health* **27**, 215–225 (2015).

93. Okewole, A. O. *et al.* Psychosis risk screening among secondary school students in Abeokuta, Nigeria: Validity of the Prodromal Questionnaire - Brief Version (PQ-B). *Schizophr Res* **164**, 281–282 (2015).

94. Razali, S. M., Abidin, Z. Z., Othman, Z. & Yassin, M. A. M. Screening for schizophrenia in initial prodromal phase: Detecting the sub-threshold psychosis. *Asian J Psychiatr* **16**, 26–31 (2015).

95. Zhang, T. *et al.* Screening schizotypal personality disorder for detection of clinical high risk of psychosis in Chinese mental health services. *Psychiatry Res* **228**, 664–670 (2015).

96. Spectral editing in proton magnetic resonance spectroscopy. Determination of GABA level in the brains of humans with ultra-high risk for schizophrenia | SpringerLink. https://link.springer.com/article/10.1007/s11172-015-1144-7.

97. Chen, F. *et al.* Identification and characterization of college students with attenuated psychosis syndrome in China. *Psychiatry Res* **216**, 346–350 (2014).

98. Solís-Vivanco, R. *et al.* Mismatch Negativity reduction in the left cortical regions in first-episode psychosis and in individuals at ultra high-risk for psychosis. *Schizophr Res* **158**, 58–63 (2014).

99. Fresán, A. *et al.* Personality features in ultra-high risk for psychosis: a comparative study with schizophrenia and control subjects using the Temperament and Character Inventory-Revised (TCI-R). *J Psychiatr Res* **61**, 168–173 (2015).

100. Tang, Y. *et al.* Prolonged cortical silent period among drug-naive subjects at ultra-high risk of psychosis. *Schizophr Res* **160**, 124–130 (2014).

101. Braham, A. *et al.* Validation of the Arabic version of the Comprehensive Assessment of At Risk Mental States (CAARMS) in Tunisian adolescents and young adults. *Early Interv Psychiatry* **8**, 147–154 (2014).

102. Üçok, A. *et al.* Cognitive deficits in clinical and familial high risk groups for psychosis are common as in first episode schizophrenia. *Schizophr Res* **151**, 265–269 (2013).

103. Mondragón-Maya, A. *et al.* Reduced P3a amplitudes in antipsychotic naïve first-episode psychosis patients and individuals at clinical high-risk for psychosis. *J Psychiatr Res* **47**, 755–761 (2013).

104. de la Fuente-Sandoval, C. *et al.* Striatal glutamate and the conversion to psychosis: a prospective 1H-MRS imaging study. *Int J Neuropsychopharmacol* **16**, 471–475 (2013).

105. Sahin, S. *et al.* The history of childhood trauma among individuals with ultra high risk for psychosis is as common as among patients with first-episode schizophrenia. *Early Interv Psychiatry* **7**, 414–420 (2013).

106. Mamah, D. *et al.* A survey of psychosis risk symptoms in Kenya. *Compr Psychiatry* **53**, 516–524 (2012).

107. de la Fuente-Sandoval, C. *et al.* Higher levels of glutamate in the associative-striatum of subjects with prodromal symptoms of schizophrenia and patients with first-episode psychosis. *Neuropsychopharmacology* **36**, 1781–1791 (2011).

108. Serrani, D. Avaliação neurocognitiva dos estados de risco ultra-alto de psicose usando a bateria MATRICS (Medição e Pesquisa de Tratamento para Melhorar Cognição na Esquizofrenia). *Archives of Clinical Psychiatry* **38**, 130–134 (2011).

109. Bulbul, O., Kurt, E., Ulasoglu-Yildiz, C., Demiralp, T. & Ucok, A. Altered Resting State Functional Connectivity and Its Correlation with Cognitive Functions at Ultra High Risk for Psychosis. *Psychiatry Research: Neuroimaging* **321**, 111444 (2022).

110. Xu, L. *et al.* Relationship Between Cognitive and Clinical Insight at Different Durations of Untreated Attenuated Psychotic Symptoms in High-Risk Individuals. *Front Psychiatry* **12**, 753130 (2021).

111. Tomyshev, A. S., Lebedeva, I. S., Omelchenko, M. A. & Kaleda, V. G. MRI-Based Markers of Changes in the Supragranular Cortical Layer in Individuals at Clinically High Risk of Endogenous Psychosis. *Bull Exp Biol Med* **171**, 483–488 (2021).

112. Zhang, T. *et al.* Progressive decline of cognition during the conversion from prodrome to psychosis with a characteristic pattern of the theory of mind compensated by neurocognition. *Schizophr Res* **195**, 554–559 (2018).
